# Harm Reduction Strategies for Thoughtful Use of Large Language Models in the Medical Domain: Perspectives for Patients and Clinicians

**DOI:** 10.2196/75849

**Published:** 2025-07-25

**Authors:** Birger Moëll, Fredrik Sand Aronsson

**Affiliations:** 1 Division of Speech, Music and Hearing, School of Electrical Engineering and Computer Science, KTH Royal Institute of Technology, Lindstedsvägen 24, Stockholm, 114 28, Sweden, 46 704851893; 2 Department of Clinical Science, Intervention and Technology, Division of Speech and Language Pathology, Karolinska Institutet, Stockholm, Sweden; 3 Theme Womens Health and Allied Health Professionals, Section of Speech and Language Pathology, Karolinska University Hospital, Stockholm, Sweden

**Keywords:** conversational AI, risk mitigation, health care innovation, assistive technology, verification protocols, governance frameworks, bias awareness, regulatory compliance, human-in-the-loop, trustworthiness, artificial intelligence

## Abstract

The integration of large language models (LLMs) into health care presents significant risks to patients and clinicians, inadequately addressed by current guidance. This paper adapts harm reduction principles from public health to medical LLMs, proposing a structured framework for mitigating these domain-specific risks while maximizing ethical utility. We outline tailored strategies for patients, emphasizing critical health literacy and output verification, and for clinicians, enforcing “human-in-the-loop” validation and bias-aware workflows. Key innovations include developing thoughtful use protocols that position LLMs as assistive tools requiring mandatory verification, establishing actionable institutional policies with risk-stratified deployment guidelines and patient disclaimers, and critically analyzing underaddressed regulatory, equity, and safety challenges. This research moves beyond theory to offer a practical roadmap, enabling stakeholders to ethically harness LLMs, balance innovation with accountability, and preserve core medical values: patient safety, equity, and trust in high-stakes health care settings.

## Introduction

Powerful large language models (LLMs) such as OpenAI’s ChatGPT series, Google’s Gemini, and others mark a significant technological shift with profound implications for various sectors, nowhere more critically than in health care [1]. Patients increasingly turn to these readily accessible models for health information, symptom checking, understanding complex medical conditions, and even emotional support related to health concerns. Simultaneously, clinical workers are exploring LLMs for tasks such as summarizing lengthy patient notes, drafting patient communications, assisting with literature reviews, generating differential diagnoses, and potentially streamlining administrative burdens [2].

While the potential benefits—democratized information access and health literacy for patients, administrative relief and decision support for clinicians—are compelling, the risks associated with using nascent, rapidly evolving, and often opaque technology in the high-stakes medical domain are substantial. Harms can range from the dissemination of inaccurate or potentially dangerous medical information with detrimental effects on patient care, to the reinforcement of biases present in training data, violations of patient privacy, degradation of critical thinking and clinical reasoning skills, and the emergence of new ethical and legal challenges regarding accountability and liability [3].

Given the widespread availability and increasing capabilities of LLMs, a purely prohibitive approach is likely impractical and risks driving usage underground, away from any institutional oversight or safety protocols. Instead, this paper advocates for a harm reduction framework. Harm reduction, traditionally applied in public health contexts such as substance use management, focuses on minimizing the negative consequences associated with certain behaviors rather than seeking solely to eliminate the behavior itself [4]. Applied to the use of LLMs in medicine, this means acknowledging their inevitable use by both patients and professionals and proactively developing strategies to make that use as safe, ethical, and beneficial as possible.

This paper, therefore, aims to identify and describe the distinct harms that LLM use may pose to patients and clinical workers, illustrate those risks through concrete examples of both problematic and thoughtful applications in clinical settings, propose harm-reduction strategies that are explicitly mapped to these scenarios and tailored to each user group, promote “thoughtful use” by encouraging critical engagement, continuous verification, and a clear awareness of model limitations, and finally, analyze the implementation challenges and ethical considerations involved in embedding these strategies within the broader health care ecosystem.

By focusing on harm reduction, we can foster a more responsible, ethical, and ultimately beneficial integration of LLMs into health care, harnessing their potential while actively mitigating the inherent risks for all stakeholders involved.

## Background

LLMs are a class of artificial intelligence models characterized by their massive size (billions of parameters) and training on vast, diverse datasets comprising text and code. Typically based on the transformer architecture, they learn complex patterns, grammar, and contextual relationships in language [5]. This enables them to perform a wide range of natural language tasks, including question answering, summarization, translation, text generation, and code writing, often with remarkable fluency.

However, their capabilities stem from predicting likely sequences of words based on statistical patterns in their training data, not from genuine understanding, reasoning, or consciousness [6]. Key limitations relevant to medicine include:

- Accuracy issues and hallucinations: LLMs can generate incorrect information (“misinformation”) or fabricate plausible-sounding but entirely false statements, citations, or data (“hallucinations”) [7]. This is particularly dangerous when providing medical advice or summarizing patient data. Research shows that hallucinations cannot be fixed and any strategy using LLMs should take into account the risk of hallucinations [8].
- Knowledge cutoffs: their knowledge is typically limited to the data they were trained on, which may be outdated, leading to incorrect advice regarding current medical guidelines, drug approvals, or treatment protocols [9].
- Bias amplification: they can inherit and amplify societal biases present in their training data (related to race, gender, socioeconomic status, age, disability, etc), potentially leading to inequitable or harmful outputs, such as differential diagnostic suggestions or biased language in generated notes [10].
- Lack of common sense and causal reasoning: they struggle with true causal reasoning, understanding implicit context, and applying common sense, which are fundamental aspects of clinical judgment. They predict correlations, not necessarily causation. Some of these issues are resolved by reasoning models [11].
- Opacity (“black box” problem): the complex internal workings of large models make it difficult, often impossible, to trace exactly why a specific output was generated. This hinders error analysis, debugging, and building trust [12].
- Opaque model versioning: when using an LLM in a graphical user interface, the model versioning is opaque, with model capabilities varying over time. Updates to system prompts can degrade performance, which recently led to excessive sycophancy [13].

It is crucial to distinguish between general-purpose LLMs (such as the public versions of ChatGPT or Gemini) and models specifically fine-tuned and rigorously validated on high-quality medical data for specific clinical tasks. The latter are still emerging and require careful regulatory oversight and transparent reporting of performance and limitations [14,15]. Even fine-tuned models are not infallible.

## Potential Harms, Challenges, and Solutions Related to LLMs in the Health Care Domain

Working with LLMs in the medical domain has many challenges. First, models need to be evaluated to assess their performance in the medical domain. Without evaluation, false confidence in model output can be potentially harmful [16]. Structured evaluation includes multiple choice questions [17], human evaluation [18], and LLM as judge [19]. In general, larger models are more performant with newer reasoning models such as DeepSeek R1 [11] and OpenAI o3 [19], showing strong performance on medical tasks. An advantage of reasoning models is that the reasoning traces can be used to improve explainability that otherwise might be lacking in LLMs [11]. Once an LLM is chosen, the next step involves improving the context of the model to increase the likelihood of an accurate result. Prompt engineering is a technique for optimizing the input text instruction that has shown potential to improve accuracy in the medical domain [20-22]. Prompt engineering techniques are helpful when information is present in the trained model, but sometimes important information can be lacking or hard to retrieve. Retrieval-augmented generation is a technique to improve the performance of LLMs by adding knowledge sources through a vector database, where additional relevant text information is retrieved whenever the LLM is called [23]. The method shows promise in the medical domain to increase accuracy and reduce hallucinations [24,25]. Another technique involves adding web browsing as a tool for the LLM so the model can search for additional medical information [26,27]. Still, context is brittle, and uneven context understanding [28,29] can lead to issues where LLMs struggle with long context. This can be especially problematic in a chat interface where the context length is not directly shown, but multiple messages can degrade performance by confusing the model by overloading the context. Finally, medical LLM agents are systems that combine these techniques to create a virtual medical agent that can simulate a medical professional through language [30,31]. Medical LLM agents have potential but are inherently risky through their more autonomous functioning that might limit human control through interventions such as human-in-the-loop [32].

## The Harm Reduction Framework

Harm reduction is a pragmatic public health philosophy and set of strategies aimed at reducing the negative consequences associated with human behaviors, particularly those deemed high-risk, without necessarily stopping the behavior itself. It originated primarily in response to harms associated with substance use, such as HIV transmission among intravenous drug users, focusing on interventions like needle exchange programs and safer sex education [33].

Applying this framework to LLMs in medicine involves acknowledging that patients and clinicians are and will continue to use these tools for health-related purposes due to their accessibility and perceived utility. The focus thus shifts from attempting outright bans to promoting safer usage patterns through targeted education, developing safety-enhancing features and clear guidelines, and fostering critical evaluation skills to minimize potential adverse outcomes such as diagnostic errors, privacy violations, reinforcing health inequities, or undermining the patient-provider relationship. For an overview of the core principles of harm reduction, see Textbox 1.

Textbox 1. **Core principles of harm reduction.**

- **Pragmatism**: accepting that risk-taking behaviors occur and focusing on minimizing resultant harm rather than solely on prevention or cessation through potentially ineffective prohibition.
- **Humanism and respect**: recognizing the dignity and rights of individuals, ensuring nonjudgmental engagement, and involving target populations (“meeting people where they are”) in strategy development.
- **Focus on harms**: prioritizing the reduction of the most significant negative consequences (health, social, or economic) associated with the behavior over symbolic gestures.
- **Balancing costs and benefits**: evaluating interventions based on their effectiveness in reducing harm relative to their potential costs or unintended negative consequences.
- **Hierarchy of goals**: recognizing that immediate, achievable goals (eg, safer use practices or information verification) may be necessary steps toward longer-term ideals (eg, optimal evidence-based decision-making).

## Illustrative Use Cases

### Overview

To make the potential harms and benefits more concrete, this section presents illustrative examples of problematic, potentially problematic, and thoughtful (harm-reduced) uses of LLMs by clinicians and patients using textboxes. The use cases are hypothetical scenarios based on observed usage patterns of LLMs and their risks.

These examples will be referenced in later sections discussing specific harms and strategies.

### Clinician Use Cases

Textboxes2^4^ present clinician use cases.

Textbox 2. **Problematic use cases for clinicians (examples to avoid).**

- **A. Diagnostic anchoring leading to missed diagnosis**: a junior doctor in a busy emergency department encounters a patient with atypical chest pain and subtle electrocardiogram (ECG) changes. Seeking quick assistance, the doctor inputs symptoms into a general-purpose large language model (LLM). The LLM generates a broad differential but highlights musculoskeletal pain and anxiety as most probable based on pattern frequency in its training data, downplaying less common but critical possibilities. Anchored by the LLM’s output, the doctor delays ordering troponins, leading to a delayed diagnosis of a non-ST elevation myocardial infarction. **Harm**: diagnostic error, delayed treatment, and potential patient harm.
- **B. Unverified automated documentation error**: a resident uses an LLM integrated into the electronic health record (EHR) to automatically generate a discharge summary for a patient with multiple comorbidities and recent medication changes. The LLM accurately summarizes most aspects but misinterprets a note about discontinuing 1 medication, instead listing it as continued. Rushed, the resident skims the summary, misses the error, and signs off. The patient’s primary care physician continues the incorrect medication post discharge, resulting in an adverse drug event requiring rehospitalization.**Harm**: patient safety risk, medication error, increased health care cost, and potential liability.
- **C. Deskilling through overreliance**: a midcareer physician finds an LLM tool adept at drafting complex consultation notes. Initially used for efficiency, the physician begins relying on it for nearly all documentation, spending less time formulating their own clinical reasoning and differential diagnoses in writing. Over several months, their ability to articulate subtle findings and complex decision-making processes independently diminishes. When faced with a particularly unusual case requiring nuanced synthesis without artificial intelligence (AI) assistance, they struggle to structure their thoughts effectively. **Harm**: deskilling, erosion of clinical reasoning skills, and potential for missed nuances in complex cases.
- **D. Compliance breach with public LLM**: a specialist is stumped by a rare presentation. To quickly gather potential insights, they use a freely available public LLM on their personal smartphone, pasting anonymized but highly specific clinical details (unique combination of symptoms, test results, and demographic hints). While seemingly anonymous, the combination of details could potentially be reidentified. This action violates institutional policy and HIPAA (Health Insurance Portability and Accountability Act) or GDPR (General Data Protection Regulation) privacy regulations regarding the use of nonapproved, nonsecure platforms for handling patient health information (PHI). **Harm**: privacy violation, institutional noncompliance, legal or professional risk, and erosion of patient trust if discovered.
- **E. Reinforcing bias in clinical notes**: an LLM, trained on historical medical records containing implicit biases, is used to help draft progress notes. When summarizing a challenging patient encounter, the LLM uses subtly different, more judgmental language to describe a patient from a minority ethnic group compared to a patient from the majority group with similar behavior, reflecting biases in its training data (eg, describing one as “uncooperative” versus the other as “expressing concerns”). The clinician, focused on medical facts, incorporates the biased language without critical review, perpetuating stereotypes within the medical record. **Harm**: bias propagation, reinforcement of health inequity, and potential impact on future care.

Textbox 3. **Thoughtful use cases for clinicians (examples to emulate).**

- **A. Efficient drafting with human oversight**: a primary care physician uses an institutionally approved, secure large language model (LLM) integrated within the electronic health record (EHR) to draft a standard referral letter to a specialist for a common condition (eg, persistent knee pain). The LLM prefills information such as patient demographics, relevant history, and current medications from structured fields. The physician meticulously reviews the draft, edits it for accuracy, adds specific clinical nuances and their precise referral question, ensures the tone is appropriate, and digitally signs the final version. **Strategy**: assistive tool within secure environment, human-in-the-loop validation, and efficiency gain for low-complexity task.
- **B. Accelerated literature review foundation**: a clinical researcher is exploring a new therapeutic area. They use a specialized medical LLM to get a broad overview of recent review papers and landmark trials, asking it to summarize key findings and methodologies. The LLM provides a structured starting point and identifies potential search terms. The researcher then uses this foundation to perform a systematic search in PubMed and other databases, retrieves the original papers identified by the LLM (and others), and critically appraises the full texts themselves, verifying the LLM’s summary and conducting their own synthesis. **Strategy**: information gathering and synthesis aid, foundation for deeper research, requires critical appraisal and verification against primary sources.
- **C. Improving clarity of nonclinical communications**: a department head needs to draft an email to staff about a new scheduling policy. They use an LLM to help structure the email, ensure a clear and concise tone, and check for grammatical errors. As this involves no patient health information (PHI) and is purely administrative, the risks are low. The final draft is reviewed for accuracy and clarity before sending. **Strategy**: low-risk use case (administrative task), communication and writing aid, efficiency.
- **D. Cocreating patient education materials**: a nurse practitioner wants to create a simplified handout explaining diabetes management for patients with low health literacy. They use an LLM to generate a first draft in plain language. Then, they \textit{carefully review and edit} the content for medical accuracy, cultural appropriateness, and clarity, potentially involving a patient focus group or health literacy expert before finalizing and approving the material through institutional channels. **Strategy**: content generation aid, requires significant human expertise for validation, and multi-stakeholder input for patient appropriateness.
- **E. Critical brainstorming for complex cases (by experts)**: a team of experienced oncologists is discussing a patient with a rare tumor type and multiple treatment failures. They use a secure, specialized LLM trained on oncological literature to brainstorm *potential* novel therapeutic combinations or clinical trials they might not have considered. They treat the output with \textit{high skepticism}, understanding it might hallucinate or misinterpret data. Any intriguing suggestion is immediately subjected to rigorous verification against primary literature, trial databases, and expert consensus before being seriously considered as a treatment option. **Strategy**: cognitive aid for experts, idea generation for outlier cases, requires extreme skepticism and mandatory verification, and not for decision making.

Textbox 4. **Potentially problematic use cases for clinicians (potential for harm).**

- **A. Rapid prerounds summarization**: an intern uses an approved electronic health record (EHR)–integrated large language model (LLM) to generate brief summaries of overnight events for their patient list just before morning rounds. It helps them quickly recall key points under time pressure. However, relying solely on the summary without also reviewing nursing notes or key alerts might cause them to miss subtle but important changes in patient status (eg, a brief desaturation event or a minor medication adjustment not flagged as critical). **Potential harm**: overlooking subtle clinical changes or incomplete information transfer. **Why potentially problematic**: useful for efficiency under pressure, but risk increases if it replaces deeper review rather than supplementing it.
- **B. Drafting standardized patient instructions**: a nurse uses an LLM tool with approved templates to draft discharge instructions for a routine procedure (eg, wound care after minor surgery). The LLM generates standard advice quickly. However, if the nurse does not carefully personalize it for the patient’s specific context (eg, limited dexterity affecting self-care, specific allergy noted elsewhere in the chart, or lack of home support), the instructions might be impractical or slightly incomplete for that individual. **Potential harm**: impractical or incomplete advice, minor patient confusion, or difficulty adhering. **Why potentially problematic**: efficient for standard tasks, generally safe, but a lack of personalization could be problematic in specific cases if not diligently checked.
- **C. Preliminary drug interaction check**: a physician quickly asks an LLM about potential interactions between 2 common medications they are considering prescribing, intending it as an initial check before consulting the definitive drug interaction database or pharmacist. The LLM might provide a correct “no major interaction found” response for common scenarios, but could miss a rare interaction, fail to account for the specific patient’s metabolism (eg, renal or hepatic function), or have slightly outdated information. **Potential harm**: false sense of security or missing a relevant interaction if subsequent checks are skipped. **Why potentially problematic**: seems like a harmless quick check, but danger lies in substituting it for authoritative tools or becoming complacent.
- **D. Generating patient communication drafts from notes**: a consultant dictates technical findings and asks an LLM integrated tool to rephrase them into simpler language for a patient portal message draft. The intention is to improve communication. However, the LLM might oversimplify complex concepts, inadvertently change the meaning, lose critical nuance, or adopt a tone that feels impersonal or does not match the clinician’s relationship with the patient. **Potential harm**: miscommunication, loss of precision, or depersonalization of communication. **Why potentially problematic**: aims to improve patient understanding, but requires very careful review and editing to ensure accuracy, completeness, and appropriate tone.

### Patient Use Cases

Textboxes5^7^ present patient use cases.

Textbox 5. **Problematic use cases for patients (examples to avoid).**

- **A. Mental health self-diagnosis and treatment delay**: a college student experiencing persistent worry, sleep problems, and difficulty concentrating asks a publicly available large language model (LLM) about their symptoms. The LLM provides information about Generalized Anxiety Disorder (GAD) and suggests common self-help strategies like mindfulness apps and exercise. Based solely on this, the student self-diagnoses GAD and tries only the suggested strategies, neglecting to consider other possibilities (eg, depression, attention-deficit/hyperactivity disorder, or thyroid issues) and delays seeking a professional assessment and evidence-based therapy for several months, during which their symptoms worsen. **Patient type**: young adult for mental health. **Harm**: misinformation (incomplete differential), delayed professional care, potential worsening of underlying condition.
- **B. Chronic condition self-management error**: a patient recently diagnosed with heart failure asks an LLM for advice on managing their condition, including diet. The LLM provides generic low-sodium advice but fails to account for the patient’s specific diuretic dosage, kidney function, or recent weight fluctuations noted by their doctor. The patient strictly adheres to the LLM’s generic advice, including increasing fluid intake as suggested for “general health,” leading to fluid overload, shortness of breath, and an avoidable emergency department visit and hospitalization. **Patient type**: chronic condition (heart failure). **Harm**: inaccurate or incomplete advice lacking personalization, direct physical harm, and avoidable health care use.
- **C. Pregnancy misinformation causing panic**: an expectant mother in her first trimester experiences mild cramping, a common occurrence. Anxious, she asks an LLM about “first trimester cramping.” The LLM, potentially hallucinating or overemphasizing rare associations from its data, includes information linking cramping to ectopic pregnancy or miscarriage without adequate context or probability weighting. The patient, lacking a medical background, panics, experiences significant distress, and makes an unnecessary urgent call to her obstetrics and gynecology doctor. **Patient type**: pregnant. **Harm**: misinformation or lack of context, undue anxiety and alarm, and potential unnecessary health care contact.
- **D. Replacing professional advice with LLM output**: an older adult with multiple chronic conditions receives a new prescription after a specialist visit but is confused about how it interacts with their existing medications. Instead of calling their pharmacist or primary care physician, they ask a general-purpose LLM. The LLM either provides outdated information about interactions (due to knowledge cutoff) or fails to grasp the complexity of the patient’s full medication list. The patient follows the LLM’s incorrect advice, leading to suboptimal treatment efficacy or an adverse interaction. **Patient type**: older adult with polypharmacy. **Harm**: nonadherence to prescribed regimen, ineffective treatment, potential adverse drug events, and bypassing safety checks.
- **E. Sharing sensitive genetic information**: a patient with a family history of a hereditary cancer syndrome receives their genetic test results. Concerned and seeking interpretation, they paste large sections of the raw genetic report, including specific mutations and personal identifiers (potentially inadvertently), into a public LLM forum or chatbot interface seeking explanations. This action exposes highly sensitive, potentially stigmatizing genetic information to a nonsecure platform with unclear data usage policies. **Patient type**: health anxiety about genetic concerns. **Harm**: critical privacy breach of sensitive genetic data, risk of data misuse, and potential for future discrimination.

Textbox 6. **Thoughtful use cases for patients (examples to emulate).**

- **A. Understanding a new diagnosis before consultation**: a patient is newly diagnosed with type 2 diabetes. Before their follow-up appointment with the diabetes educator, they use a large language model (LLM) to ask clarifying questions such as “Explain HbA_1c_ in simple terms,” “What are common first treatments for type 2 diabetes?," and “What lifestyle changes help manage blood sugar?" They compile a list of specific questions based on the LLM’s answers to ask the educator, ensuring they understand the nuances and personalized aspects from the professional. **Patient type**: newly diagnosed chronic condition. **Strategy**: health literacy aid, preparing informed questions for consultation, using LLM for background, and verifying or personalizing with provider.
- B**. Finding credible mental health resources**: someone experiencing persistent low mood and lack of motivation asks an LLM to “List reputable organizations providing information and support for depression in [their country/region].” The LLM generates names such as national mental health charities, government health websites, and professional psychological associations. The user then visits the official websites of these organizations to verify their credibility, explore their resources, and find contact information for helplines or provider directories. **Patient type**: mental health concerns. **Strategy**: resource finding aid (identifying potential sources) and requires user verification of source credibility.
- **C. Preparing questions for prenatal visits**: an expectant parent uses an LLM to generate a list of relevant questions to ask their midwife at their upcoming 20-week appointment, based on typical milestones and screenings discussed at that stage. Prompts such as “What questions should I ask my midwife at the 20-week prenatal visit?” help them organize their thoughts and ensure they cover key areas during the limited appointment time. **Patient type**: pregnant. **Strategy**: preparing for consultation, organizing thoughts, and facilitating communication with provider.
- **D. Learning about recommended health screenings**: a healthy adult wants to understand current preventive health recommendations. They ask an LLM: “According to the USPSTF, what are the recommended cancer screenings for a 55-year-old male with no family history?” The LLM provides a summary (eg, colorectal or lung if smoking history). The patient uses this information as a starting point for discussion with their doctor, cross-referencing with trusted sources such as the Centers for Disease Control and Prevention (CDC) or official United States Preventive Services Task Force (USPSTF) website. **Patient type**: general or preventive health. **Strategy**: information seeking on guidelines, basis for doctor discussion, and requires verification against authoritative sources.
- **E. Deciphering medical terminology in reports**: a patient receives access to their blood test results or an imaging report through a web-based portal and encounters unfamiliar medical terms (eg, “glomerular filtration rate” and “hypodense lesion”). They use an LLM to get simplified definitions of these specific terms within the context provided. This helps them better understand the report before their follow-up appointment, enabling them to ask more targeted questions to their clinician about the findings’ significance. **Patient type**: general or chronic condition follow-up. **Strategy**: health literacy aid (terminology explanation), facilitates understanding of personal health data, and supports preparation for clinician discussion.

Textbox 7. **Potentially problematic use cases for patients (potential for harm).**

- **A. Looking up medication side effects**: a patient prescribed a new antidepressant uses a large language model (LLM) to “list the side effects of [medication name].” The LLM generates a comprehensive list, including common, uncommon, and rare side effects, often without clear indication of frequency or severity. While informative, reading about numerous severe but rare side effects might cause the patient significant anxiety (nocebo effect) and potentially lead to nonadherence even before trying the medication. **Potential harm**: undue anxiety, nocebo effect, and premature nonadherence. **Why potentially problematic**: seeking information is reasonable, but LLM output often lacks context (frequency, severity, or personal risk factors) needed for balanced understanding.
- **B. Exploring alternative therapies**: a patient diagnosed with chronic back pain, partially unsatisfied with conventional options, asks an LLM about “alternative therapies for chronic back pain.” The LLM lists options ranging from evidence-supported (eg, yoga or acupuncture for some) to unproven or pseudoscientific (eg, specific supplements or energy healing methods) without clearly distinguishing the level of evidence or potential risks. **Potential harm**: pursuing ineffective or costly therapies, delaying evidence-based care, and potential harm from unregulated treatments. **Why potentially problematic**: exploration is understandable, but LLM output often fails to provide necessary quality filtering, evidence grading, or safety warnings.
- **C. Interpreting laboratory results before discussion**: a patient sees their cholesterol panel results in their web-based portal before their doctor’s appointment. They input the numbers into an LLM asking, “What does an LDL of 140 mg/dL mean?” The LLM provides a generic explanation of LDL levels and risk categories. This might give the patient a basic understanding but could lead them to misinterpret their personal risk without considering other factors (age, blood pressure, diabetes status, or family history) or feel falsely reassured or alarmed. **Potential harm**: misinterpretation of personal risk, undue anxiety, or false reassurance based on incomplete context. **Why potentially problematic**: patient initiative to understand data is positive, but LLM interpretation lacks the personalization essential for clinical meaning.
- **D. Seeking diet plans for a medical condition**: a patient newly diagnosed with irritable bowel syndrome (IBS) asks an LLM to “create a low-FODMAP diet plan.” The LLM generates a sample plan that seems helpful. However, it might lack crucial details about the phased approach (elimination or reintroduction), portion sizes, specific food triggers that vary individually, or the importance of working with a dietitian for personalization and nutritional adequacy. **Potential harm**: incorrect implementation of a complex diet, nutritional deficiencies, unnecessary restrictions, and frustration. **Why potentially problematic**: useful for initial ideas, but lacks the detailed guidance and personalization required for effective and safe implementation of therapeutic diets.

## Potential Harms of LLMs in the Medical Domain

The use of LLMs for medical information or tasks introduces distinct risks, which can differ in nature and severity depending on whether the user is a patient or a clinical professional. The examples in the current section illustrate many of these dangers.

### Harms Primarily Affecting Patients

Patients often interact with LLMs without formal training, established validation processes, or a deep understanding of the technology’s limitations, making them particularly vulnerable to various harms, as illustrated in the patient use cases.

- Misinformation and inaccuracy: receiving factually incorrect, incomplete, or outdated medical information is a primary risk. This can range from incorrect descriptions of conditions to unsafe advice on self-treatment or medication use, potentially leading to suboptimal health decisions [1]. The heart failure self-management error (B in Textbox 5) exemplifies this danger.
- Hallucinations creating false realities: LLMs may generate entirely fabricated medical “facts,” studies, or treatment options that sound plausible but have no basis in reality [7]. This can cause significant confusion, anxiety, or lead patients down dangerous paths based on nonexistent information.
- Delayed or inappropriate care seeking: overreliance on seemingly authoritative LLM advice might lead patients to delay seeking necessary professional medical attention or to seek the wrong type of care. The mental health self-diagnosis scenario (A in Textbox 5) illustrates how this delay can occur, potentially allowing conditions to worsen.
- Misinterpretation of complex or nuanced information: patients may struggle to correctly interpret the nuances, context, or limitations of LLM-provided information, especially concerning statistics, risk factors, or complex treatment regimens. This can lead to misunderstandings or incorrect application of advice.
- Critical privacy risks and data security breaches: inputting detailed personal health information (PHI), symptoms, or concerns into publicly accessible or poorly secured LLM platforms poses significant privacy risks. This data could be stored, used for training, or breached, potentially enabling reidentification or discrimination [34], as highlighted by the genetic data sharing example (E in Textbox 5).
- Exposure to and perpetuation of bias leading to health inequity: receiving medical advice or information that reflects societal biases embedded in the LLM’s training data can reinforce stereotypes related to race, gender, age, or socioeconomic status, potentially leading to inequitable self-care decisions or interactions with the health care system [35,36].
- False reassurance or unjustified alarm: LLM responses might inaccurately reassure a patient about serious symptoms, leading to delayed care, or conversely, cause undue panic over minor issues by highlighting rare worst-case scenarios without proper context, as seen in the pregnancy misinformation case (C in Textbox 5).
- Undermining the patient-provider relationship: patients may bring LLM printouts to appointments, and if the information conflicts with professional advice or is presented confrontationally, it can strain trust and communication with health care providers.

### Harms Primarily Affecting Clinical Workers

Clinicians might use LLMs seeking efficiency or decision support but face risks related to professional standards, diagnostic accuracy, workflow integration, legal responsibilities, and ethical practice, as illustrated in the current section and Textboxes 2-4.

- Diagnostic errors and compromised clinical judgment: overreliance on LLM outputs (automation bias) for differential diagnoses or interpreting results can lead to diagnostic errors through anchoring, premature closure, or overlooking critical nuances in the patient’s presentation [37]. The missed non-ST elevation myocardial infarction case (A in Textbox 2) provides a stark example. This undermines the nuanced clinical reasoning process.
- Deskilling and erosion of core clinical competencies: frequent delegation of cognitive tasks such as summarizing information, drafting notes, or generating differentials to LLMs may, over time, erode the clinicians’ own skills in these areas, potentially leading to reduced proficiency, as illustrated in the deskilling scenario (C in Textbox 2).
- Workflow integration burdens and validation challenges: safely integrating LLM use into time-pressured clinical workflows requires robust validation steps. Failure to properly review and verify LLM output, as seen in the discharge summary error (B in Textbox 2), can introduce critical errors with serious patient safety consequences.
- Liability and accountability vacuum: determining legal and professional responsibility when an LLM contributes to a diagnostic error, treatment mistake, or privacy breach is complex and currently lacks clear legal precedent [38]. This ambiguity poses significant risks to clinicians and health care institutions.
- Data security breaches and regulatory noncompliance: using LLMs, particularly publicly available, nonenterprise versions, for tasks involving PHI can lead to violations of privacy regulations such as HIPAA (Health Insurance Portability and Accountability Act) or GDPR (General Data Protection Regulation) [30]. The example of using a public LLM for case insights (D in Textbox 2) highlights this compliance risk and potential loss of patient trust.
- Propagation and institutionalization of bias: uncritically accepting or incorporating LLM-generated text (eg, patient notes or communication drafts) that contains subtle biases inherited from training data can embed these biases within patients’ records and institutional practices [31,36]. The biased clinical notes example (E in Textbox 2) shows how this can occur, potentially affecting care quality and equity.
- Communication barriers and depersonalization of care: relying heavily on LLM-generated text for patient communication might lead to less empathetic, overly standardized interactions, potentially hindering the development of rapport and trust central to the patient-provider relationship.
- Cost, resource allocation, and opportunity costs: implementing medically validated, secure, enterprise-grade LLM solutions requires significant investment. Focusing resources on LLMs might divert funding from other essential health care services or workforce needs.

### Comparative Risk-Magnitude Synthesis

Table 1 translates the qualitative harms into an ordinal 0‐10 scale that weights prevalence and severity equally. Prevalence (reported error or breach frequency) and severity (clinical or legal consequences) were weighted equally, then mapped to the 0‐10 scale. Where multiple studies diverged (eg, hallucination 1% versus 60%), the higher-risk context was used, reflecting real-world open-ended usage. Scores are ordinal, not interval meant to prioritize mitigation effort, not to imply precise quantification. Three patterns stand out.

**Table 1.** Quantitative risk matrix for patient- and clinician-facing harms of medical LLM^a^ use.

Risk category (definition)	Exemplary evidence	Magnitude of patients, n	Rationale of patients	Magnitude of clinicians, n	Rationale of clinicians
Misinformation or hallucination (incorrect, fabricated, or outdated medical content)	Hallucination rates range from ≈1%‐2% in highly constrained note-taking tasks to >60% on open-ended clinical vignettes [39]	8	Directly shapes self-care decisions	7	Anchoring and automation bias during diagnosis
Diagnostic error and overreliance (LLM suggestions misdirect clinical reasoning or patient self-triage)	ChatGPT overprescribed imaging or antibiotics and mis-triaged ED^b^ cases [40]	7	Delay or inappropriate help-seeking	8	Missed or unnecessary care or liability exposure
Bias and inequity (outputs reflecting racial, ethnic, or socioeconomic bias)	Measurable race-linked content distortions across 4 leading LLMs [41]	6	Unequal advice or trust erosion	6	Biased notes or unequal treatment plans
Privacy or data security (exposure or secondary use of protected health information)	133 million US health records breached in 2023; most occurred via cloud APIs^c^ and vendor platforms [42]	9	Identity and discrimination risk	8	Regulatory fines or reputational harm
Deskilling or competency erosion (reduced practice of core cognitive skills)	Mixed-method review flags “erosion of diagnostic expertise over time” when clinicians over-delegate to AI^d^ [43]	—^e^	Not applicable	7	Long-term skill atrophy or dependence
Care-pathway disruption (delays, overuse, or inappropriate self-management)	ED study above, plus case reports of delayed oncology care after ChatGPT reassurance [40]	7	False reassurance or unnecessary alarm	6	Workflow overload or excess testing
Relationship or trust erosion (conflicting advice or anti-AI bias)	Parents rated ChatGPT content more trustworthy than pediatric experts (n=116) [44]; other surveys show anti-AI bias if use is disclosed [45]	6	Preferential trust in AI can undermine provider rapport	5	Skepticism when AI involvement is revealed
Liability or accountability gap (unclear legal responsibility when AI harms occur)	Ongoing debate on “who pays if AI goes rogue” [46]; malpractice lawyers anticipate novel claims [47]	4	Limited direct recourse	8	Uncertain standard of care or rising legal exposure

a LLM: large language model.

b ED: emergency department.

c API: application programming interface.

d AI: artificial intelligence.

e Not available.

### High-Priority, Bidirectional Risks

Misinformation or hallucination (patients=8 and clinicians=7) and privacy or data-security (patients=9 and clinicians=8) dominate the landscape. Empirical hallucination rates above 60% in open-ended clinical tasks [39] and the continued surge of cloud-based health data breaches encompassing >130 million US records in 2023 [42] justify near-maximal scores. As both patients and clinicians rely on textual output but lack perfect visibility into model provenance, these categories merit the most stringent guardrails: persistent disclaimers, retrieval-augmented prompts, and enterprise sandboxing with end-to-end encryption.

### Asymmetric Professional Exposure

Diagnostic error or overreliance (7 vs 8) and liability or accountability (4 vs 8) skew toward clinicians. Emergency department simulations show that ChatGPT overorders antibiotics and imaging while missing critical triage cues [40], and malpractice experts warn of an “artificial intelligence (AI)–driven grey zone” for standard-of-care determinations [46,47]. These findings reinforce the paper’s insistence on “human-in-the-loop” validation and clear institutional ownership of AI-assisted decisions.

### Long-Term Systemic Concerns

Deskilling receives a low patient score (2) but a relatively high clinician score (7) after mixed-method evidence that routine delegation erodes diagnostic acumen over time [43]. Bias or inequity sustains midrange scores (6, 6): quantitative audits reveal consistent racial and socioeconomic distortions in leading LLMs [41]. Although the immediate clinical impact may be subtler than overt misinformation, these insidious effects accumulate, necessitating periodic bias audits, diversified training corpora, and ongoing skills-maintenance programs.

Overall, the numeric synthesis underscores that not all harms are equal or evenly distributed between user groups. Prioritizing mitigation resources toward the highest-scoring, cross-cutting risks while tailoring secondary interventions to group-specific exposures operationalizes the harm-reduction framework proposed in this paper.

### Harms Related to Nonuse of LLMs

A way to classify the relative risk of LLM use is to compare use to nonuse. Without LLMs, health care workers can use electronic tools such as search engines, websites, and internal documents for information retrieval. This approach is likely slower than the use of LLMs, although the information has the potential to be more accurate. In a scenario with a shortage of health care workers, nonuse of LLMs might lead to less information retrieval by health care workers, which over time can lead to errors in diagnosis and less knowledge gained by health care workers. For patients, nonuse can lead to less nuance in the understanding of their own symptoms because standard websites for health information might lack the knowledge to answer questions that involve several symptoms. For instance, using LLMs to ask a question about the risk of having a cold during pregnancy gives a nuanced example with information about symptoms and when to contact a health care provider [48]. This information is not immediately present when using a web search for the query [49]. There are several articles about individuals using LLMs to diagnose medical conditions, including Hodgkin lymphoma [50], thyroid cancer [51], and tethered cord syndrome in a child aged 4 years [52]. The last case is noteworthy because 17 doctors failed to diagnose the disorder [52]. Although these cases are anecdotal, they are still noteworthy. A key insight is that the individual with the medical condition has the best ability to describe their symptoms. Using an LLM is several orders of magnitude cheaper than traditional health care, and a user can consume much more health care through talking to an LLM, as long as they have notable symptoms they can describe. Here, nonuse would mean that these conditions went undiagnosed or were diagnosed at a later date, which could be harmful or even deadly to patients. In line with findings from the National Academy of Medicine report on Artificial Intelligence in Medicine, we argue that the benefits of LLMs in health care vastly outweigh the inherent risks [53]. We believe that a harm reduction approach is the best way to reduce the risks while maintaining the benefits of the use of the technology.

## Harm Reduction Strategies for Thoughtful Use

**Figure 1.**
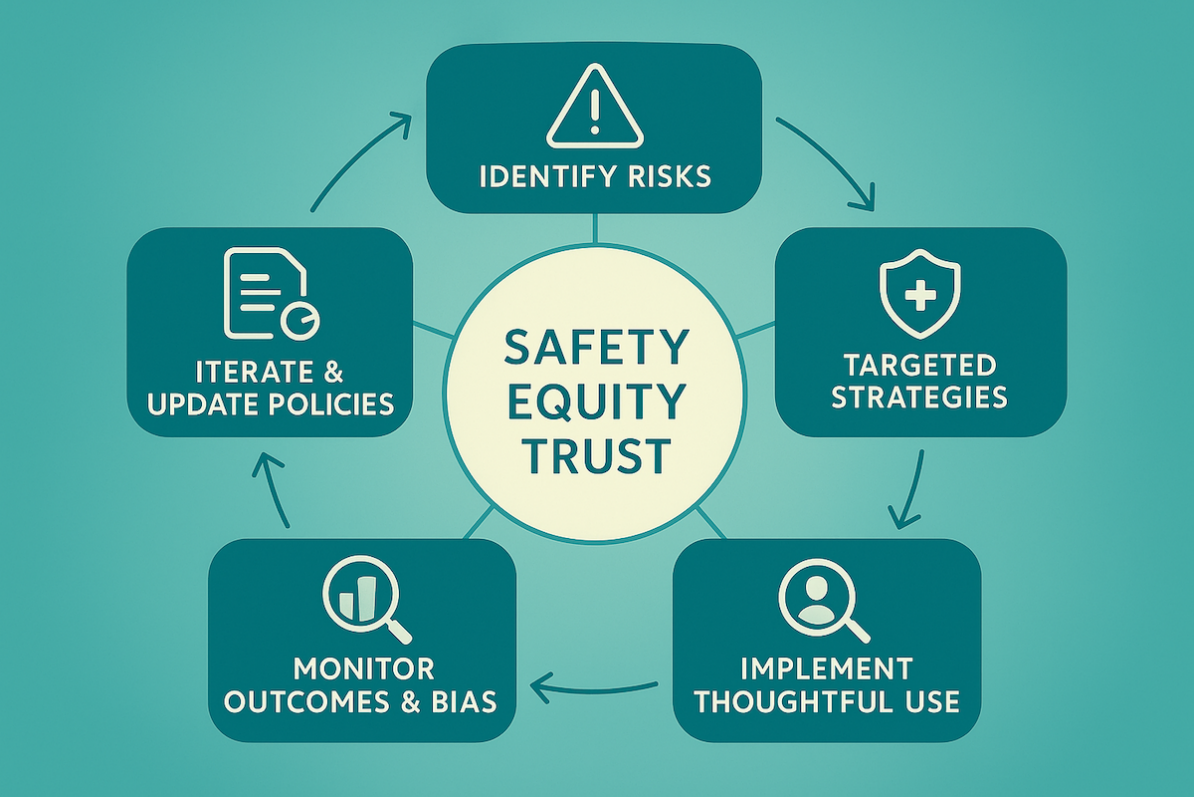
Cycle of thoughtful use of LLMs in medicine. LLM: large language model.

A harm reduction approach necessitates distinct but complementary strategies for patients and clinicians, focusing on education, promoting critical usage patterns, implementing technical and procedural safeguards, and fostering transparency. The thoughtful use cases exemplify many of these strategies in action (see Figure 1 for a conceptual overview and Table 2 for specific examples).

**Table 2.** Patient harms and mitigating strategies.

Harm affecting patients	Mitigating strategies for patients
Misinformation and inaccuracy	Clear disclaimers and contextual warnings Promotion of verification habits Safer-prompting guidance Output transparency and source attribution Health-literacy support Safe-use guide (Multimedia Appendix 1)
Hallucinations creating false realities	Clear disclaimers and contextual warnings Promotion of verification habits Safer-prompting guidance Health-literacy support Safe-use guide (Multimedia Appendix 1)
Delayed or inappropriate care seeking	Clear disclaimers and contextual warnings Promotion of verification habits Safer-prompting guidance Safety-oriented UI/UX^a^ design Safe-use guide (Multimedia Appendix 1)
Misinterpretation of complex or nuanced information	Health-literacy support Safer-prompting guidance Output transparency and source attribution Safe-use guide (Multimedia Appendix 1) Plain-language AI^b^ glossary (Multimedia Appendix 2)
Critical privacy risks and data-security breaches	Privacy and data-use transparency Safe-use guide (Multimedia Appendix 1—privacy checklist)
Exposure to and perpetuation of bias leading to health inequity	Promotion of verification habits Health-literacy support Output transparency
False reassurance or unjustified alarm	Clear disclaimers and contextual warnings Promotion of verification habits Safer-prompting guidance Safe-use guide (Multimedia Appendix 1)
Undermining the patient-provider relationship	Safer-prompting guidance Promotion of verification habits Safe-use guide (Multimedia Appendix 1)

a UI/UX: user interface/user experience.

b AI: artificial intelligence.

### Strategies for Patients

Empowering patients to interact with LLMs more safely involves increasing their awareness, providing tools for critical assessment, and managing expectations.

- Ubiquitous, clear disclaimers, and contextual warnings: LLM interfaces providing health-related information should persistently display prominent disclaimers stating the output is not medical advice, may be inaccurate or incomplete, and is not a substitute for professional consultation.
- Actively promoting verification habits: interfaces should actively encourage and facilitate verification. This could involve prompts such as “Consult your doctor about this information” or integrating links to reputable sources (eg, Centers for Disease Control and Prevention, National Institutes of Health, and World Health Organization) for cross-referencing, though source attribution in current LLMs is often poor. The patient learning about screening (C in Textbox 6) or finding mental health resources (B in Textbox 6) exemplifies the desired user behavior of verification.
- Guidance on safer prompting techniques: educating users or providing interactive guidance, potentially by a health care worker on how to frame questions for informational purposes (eg, “Explain X” or “List resources for Y”) rather than diagnostic or treatment requests (“Do I have Z?“ or “Should I take medication A?"). Using LLMs to prepare questions for a doctor (A in Textbox 6) is a safer prompting strategy.
- Enhancing output transparency and source attribution: where technically feasible, LLMs should ideally indicate the sources of their information or provide confidence scores for their statements. This is currently a major technical challenge, but a key goal for safer AI.
- Transparency on privacy and data use: provide clear, accessible information about how patient queries and data are stored, used (especially for model training), and protected. Offer simple mechanisms for users to manage or delete their data. This addresses risks seen in the genetic data sharing example (problematic use case E in Textbox 5).
- Safety-oriented user interface and user experience design: design interfaces that visually differentiate LLM output from human advice, avoid overly anthropomorphic or authoritative presentations, and make safety warnings and limitations impossible to ignore.
- Integrating health literacy support: connect users with general health literacy resources to help them better understand medical concepts and critically evaluate information from any source, including LLMs. Using an LLM to decipher terminology (E in Textbox 6) aligns with this.

A guide for safe patient use taking into account these key points is attached as Multimedia Appendix 1.

### Strategies for Clinical Workers

For clinicians, harm reduction focuses on responsible, ethical, and effective integration of LLMs into professional workflows through structured training, clear governance, robust validation processes, and appropriate technical environments.

- Comprehensive, mandatory training and competency development: implement mandatory, role-specific training covering LLM capabilities, limitations (accuracy, bias, knowledge cutoffs, or hallucinations), ethical use, privacy regulations, institutional policies, and critically, techniques for validating LLM output [2]. Competency assessments should be considered.
- Establishing clear institutional use cases and risk-stratified guidelines: health care organizations must develop, disseminate, and enforce clear policies defining approved LLM tools, permitted use cases (eg, drafting noncritical notes and literature search) versus high-risk or prohibited uses (eg, autonomous diagnosis or treatment planning), and required safeguards for each [54]. These guidelines need to be regularly updated.
- Relentlessly emphasizing the indispensable “human-in-the-loop”: continuously reinforce the nonnegotiable principle that LLMs are assistive tools only. All clinically relevant LLM output must be reviewed, critically evaluated, edited, and ultimately validated by a qualified human clinician who takes full responsibility. The thoughtful referral letter drafting example (A in Textbox 3) embodies this.
- Developing and implementing robust validation and cross-referencing protocols: create practical, standardized procedures for clinicians to verify LLM-generated clinical information against authoritative sources (eg, current guidelines, primary literature, or electronic health record data). This is crucial for tasks such as literature review or brainstorming complex cases (E in Textbox 3).
- Clear guidelines on transparency with patients regarding LLM use: establish explicit institutional policies on if, when, and how clinicians should disclose the use of LLM assistance in patient care or communication, maintaining transparency and trust.
- Mandating technical and administrative safeguards within secure environments: prioritize secure, enterprise-grade, HIPAA or GDPR–compliant LLM platforms that are centrally procured, configured, and monitored by the institution. Make the sanctioned tool the path of least resistance by integrating it directly into electronic health records and other clinical workflows, enabling single sign-on, and supplying clear usage guides, templates, and training. Role-based access controls, end-to-end encryption, detailed audit logs, and continuous security monitoring should be enabled by default, so clinicians can work with PHI confidently and conveniently on the approved platform.
- Promoting active bias detection and mitigation practices: train clinicians to actively scrutinize LLM outputs for potential biases (related to demographics, language, etc) and to consciously mitigate these biases during review and editing, preventing their propagation as seen in the problematic note-drafting example (problematic use case E in Textbox 2). Tools for bias detection could be integrated into the review process.
- Fostering continuous interdisciplinary oversight and governance: establish institutional committees (including clinicians, information technology, legal, ethics, or patient representatives) to oversee LLM implementation, monitor outcomes, review incidents, update policies, and ensure alignment with organizational values and patient safety goals.
- Evaluation of LLMs with prompts for specific clinical tasks: assign clinical workers to evaluate specific LLMs with specific prompts and create a document of verified prompts and model versions that have been tried by clinical workers with descriptions of safe use. An example of an evaluation form is available in Multimedia Appendix 3.
- Anonymous surveys of LLM use: in line with the strategy of harm reduction, we advocate for open, anonymous discussions about the use of LLMs within the medical context, even when this use could be in breach of regulation. By allowing medical staff to anonymously give information and discuss what models they use, how well they work, and prompts to use to improve performance, this can reduce harm, similar to how needle exchange programs [55] are a sometimes controversial but useful prevention strategy for HIV. Similarly, these anonymous surveys can be a great way to inform medical professionals of best practice use to reduce harm (using best-performing models in the specific medical context, proper prompting techniques, adding clinical sources, avoiding uploading personal information, and double-checking results with a trusted source). An example of an anonymous survey is found in Multimedia Appendix 4.

Both strategies for thoughtful use by patients and clinicians are dependent on both regulators and developers. Contextual warnings, provided sources, and advice to seek out external validation are dependent on the tools used. Decisions regarding what platforms are compliant and how to work with clinical data are based on current health care regulations. For strategies for mitigating harms for clinicians, see Table 3.

**Table 3.** Clinician harms and mitigating strategies.

Harm affecting clinicians	Mitigating strategies
Diagnostic errors and compromised clinical judgment	Mandatory training and competency development Clear institutional use cases and risk-stratified guidelines “Human-in-the-loop” emphasis Robust validation and cross-referencing protocols LLM^a^-prompt evaluation (Multimedia Appendix 3) Foundational curriculum (Multimedia Appendix 5) Safeguarding clinical acumen (Multimedia Appendix 6) Incident or near-miss reporting form (Multimedia Appendix 7) Institutional LLM governance committee (Multimedia Appendix 8)
Deskilling and erosion of core clinical competencies	Mandatory training and competency development “Human-in-the-loop” emphasis Safeguarding clinical acumen (Multimedia Appendix 9) Foundational curriculum (Multimedia Appendix 5)
Workflow integration burdens and validation challenges	Clear institutional use cases and risk-stratified guidelines Robust validation and cross-referencing protocols Technical and administrative safeguards in secure environments LLM-prompt evaluation (Multimedia Appendix 3) Clinician-ready prompt library (Multimedia Appendix 2) Institutional LLM governance committee (Multimedia Appendix 8)
Liability and accountability vacuum	Clear institutional use cases and risk-stratified guidelines “Human-in-the-loop” emphasis Transparency guidelines for patients Continuous interdisciplinary oversight and governance Institutional LLM governance committee (Multimedia Appendix 8)
Data-security breaches and regulatory noncompliance	Mandatory training and competency development Clear institutional use cases and risk-stratified guidelines Technical and administrative safeguards in secure environments Institutional LLM governance committee (Multimedia Appendix 8) Anonymous LLM-use surveys (Multimedia Appendix 4)
Propagation and institutionalization of bias	Mandatory training and competency development Active bias-detection and mitigation practices LLM-prompt evaluation (Multimedia Appendix 3) Foundational curriculum (Multimedia Appendix 5) Institutional LLM governance committee (Multimedia Appendix 8)
Communication barriers and depersonalization of care	Mandatory training and competency development “Human-in-the-loop” emphasis Transparency guidelines for patients Foundational curriculum (Multimedia Appendix 5)
Cost, resource allocation, and opportunity costs	Clear institutional use cases and risk-stratified guidelines Continuous interdisciplinary oversight and governance Institutional LLM governance committee (Multimedia Appendix 8)

a LLM: large language model.

## Discussion

### Principal Findings

Our analysis shows that LLMs are already influencing both lay and professional health behavior, often outside formal oversight. A harm-reduction lens, adapted from public health practice, offers a pragmatic path between prohibition and uncritical adoption. For patients, the priority is to transform passive consumption of model output into a critically verified information-seeking process that preserves privacy and promotes timely care-seeking. For clinicians, the core insight is that LLMs can safely augment but never replace clinical judgment when they are used within secure, governed environments that mandate human verification, bias checks, and transparent disclosure. Implementing these measures demands institution-wide policies, continuous training, and interdisciplinary oversight but can preserve patient safety, equity, and trust while unlocking administrative efficiencies and decision-support benefits.

### Comparison to Prior Work

Earlier commentaries have cataloged the technical limitations of LLMs, hallucinations, knowledge cutoffs, and bias and called for caution [1-3,7,10,14]. Our work extends this literature in 3 ways. First, we delineate harms and mitigations separately for patients and clinicians, recognizing their distinct agency, expertise, and risk profiles. Second, we translate abstract “safe AI” principles into actionable tools (eg, clinician-patient risk matrix and governance checklists) that can be embedded in existing workflows. Third, by explicitly importing harm-reduction theory from substance-use policy [4,33], we reframe LLM governance as a continuum of safer-use practices rather than a binary of use versus ban, a perspective largely absent from prior medical-AI discourse.

### Handling Health Care Stakeholders

Patients, clinicians, developers, and regulators have different viewpoints on the use of LLMs in health care that needs to align for safe deployment. A key issue is shadow use of LLMs, where patients and clinicians use LLMs without approval from regulators. In this scenario, regulators and developers could be misaligned because problematic use can shape public opinion and later regulation. LLM developers could offer weak safeguards against the use of LLMs in a clinical domain to comply with regulations while benefiting from increased usage in the domain. Likewise, patients and clinicians can be misaligned, where patients can gain a sense of autonomy and a perceived sense of health understanding through the use of LLMs that can limit contact with health professionals. Regulators and health professionals can be misaligned if the use of LLMs can be perceived as helpful for the diagnosis and treatment of patients but is prohibited. This can lead to problematic use where health professionals do not disclose that patient diagnoses were done without human oversight. The lack of access to hardware to run LLMs locally through GPUs is another key concern. Larger models are more performant but might realistically only be available through an API, where patient data is sent to another server located elsewhere. This is a potentially serious issue because the regulation regarding health data might inadvertently lead to worse performance of LLMs in the health care domain. Here, stakeholders need to balance health data privacy rules with the need for performant models.

### Mitigating Stakeholder Misalignment

To ensure the successful and ethical integration of LLMs, it is imperative to bridge the differing perspectives of patients, clinicians, developers, and regulators. Transparent dialogue, open forums for addressing concerns, and shared development of guidelines can help align these stakeholders and create synergies based on the knowledge within each group. Safe experimentation, using synthetic data when necessary to protect patient privacy, allows health care professionals to test LLMs in controlled environments. Education on LLM capabilities and limitations is crucial for all parties, enabling patients to make informed decisions about their health information and clinicians to confidently use these tools. By fostering a collaborative environment and prioritizing open communication, we can minimize misalignment and maximize the safe and beneficial adoption of LLMs in health care.

### Do We Really Need a Human-in-the-Loop?

Research shows that the time saved by use of LLMs might be reduced by the time needed to verify outputs [56]. As such, human-in-the-loop systems where medical professionals check outputs from LLM systems might risk reducing the time saved by their use. Still, we argue that human-in-the-loop is a sensible default for the use of LLMs in the health care sector. As AI systems improve, we believe that autonomous LLM systems will be introduced in the health care sector. Many tasks that we today leave to computers, such as counting, were previously done by humans for control. However, LLMs are not calculators, and caution should be advised. By advocating for human-in-the-loop as the baseline, we can guarantee safe deployments of AI systems in the medical domain. Once these systems are deployed and work well, evaluation can become more automated. This approach is also in line with how work might change through AI in other sectors. Many occupations might have tasks involving the orchestration and evaluation of LLMs and LLM agents. As such, human-in-the-loop seems like a sensible, safe way forward.

### The Twin Goals of LLM Literacy and Health Literacy

When LLMs become a part of standard treatment for patients, for instance, as support for individuals who recently received a diagnosis of a chronic illness, they can be used to facilitate 2 important goals. First, to improve the LLM literacy both for clinicians, who build the LLM-based treatments, and for patients who take part in these treatments, and second, to improve health literacy both for patients and clinicians. For clinicians, health literacy can be seen as their ability to diagnose and solve health problems, while for patients, it is the patient’s own ability to diagnose and solve their own health problems with help from the health care sector. By improving LLM literacy and health literacy, we can reduce risks related to LLM use and improve health decisions among a large portion of the public. This is in line with the National Academy of Medicine report on Artificial Intelligence in Medicine that highlights patient education and engagement through LLMs as an important use case and proficiency in using LLMs among clinicians as a key to successful use [53].

### Navigating Patient Autonomy and LLM-Informed Diagnostic Exploration

A particularly complex facet of LLM integration in health care revolves around patient autonomy and the increasing tendency for individuals to use these tools for diagnostic exploration before, or sometimes instead of, seeking professional medical assessment. The reality, as highlighted by examples of patients attempting self-diagnosis for mental health or chronic conditions (Textboxes1, 3,  4), is that readily accessible LLMs empower patients to investigate their symptoms and potential conditions independently. This behavior stems from various factors aligned with the principle of patient autonomy: a desire for knowledge and understanding, efforts to overcome access barriers such as wait times or cost, convenience, and sometimes dissatisfaction with prior health care encounters. As LLM capabilities continue to advance, particularly those models incorporating reasoning [11], their use for preliminary self-assessment is likely to become even more prevalent.

From a harm reduction perspective, simply prohibiting or dismissing this patient’s behavior is neither feasible nor respectful of autonomy. Instead, the health care system must adapt, acknowledging that patients will increasingly arrive at consultations armed with LLM-generated hypotheses about their conditions. The critical challenge lies in channeling this patient engagement constructively while mitigating the significant risks associated with unguided, LLM-based self-diagnosis. These risks are substantial, including anchoring on inaccurate information, overlooking serious conditions due to incomplete data input or LLM limitations (lack of physical examination, nuanced history, or contextual understanding), experiencing undue anxiety from misinterpreted or alarming outputs (as seen in the pregnancy example, see Textbox 5), delaying necessary care based on false reassurance, or even initiating inappropriate self-treatment based on flawed LLM suggestions [2,3]. Furthermore, LLM outputs can perpetuate biases, potentially leading patients to misinterpret their symptoms through a biased lens before even speaking to a professional [35,36].

The most productive path forward involves reframing the clinical encounter. Rather than viewing LLM-derived information as a threat to clinical authority, it should be seen as a potential, albeit imperfect, starting point for a collaborative diagnostic conversation.

### Templates for Clinicians and Patients

To further translate the harm reduction principles discussed in this paper into tangible, operational practices, we have developed a suite of actionable templates and frameworks, detailed in MultimediaAppendices 1^10^. These resources are designed to support various stakeholders in the safe and effective integration of LLMs into health care environments.

For clinicians, these tools aim to enhance understanding, improve practice, and ensure safety. Multimedia Appendix 4 offers an anonymous survey instrument to gather insights into current LLM use and safety practices among staff. Multimedia Appendix 3 provides a structured evaluation log for systematically assessing specific LLM prompts and versions for clinical tasks. A foundational curriculum for clinician training is outlined in Multimedia Appendix 5, while Multimedia Appendix 9 details strategies for safeguarding clinical acumen against deskilling. To aid daily practice, Multimedia Appendix 6 presents a prompt library with clinician-ready starters. Furthermore, Multimedia Appendix 7 offers a standardized LLM-related clinical incident and near-miss reporting form to facilitate learning from real-world events.

For patients, the appendices focus on empowerment through understanding and safe usage. Multimedia Appendix 1 is a comprehensive guide on “Using AI Chat Tools for Health,” designed to help patients navigate these tools safely. To improve LLM literacy, Multimedia Appendix 2 provides a plain-language AI glossary of common terms.

At an institutional level, Multimedia Appendix 8 details a framework for an institutional LLM governance committee, outlining its structure and mandate to ensure robust oversight. Many of the clinician-focused appendices, such as the survey Multimedia Appendix 4, evaluation log Multimedia Appendix 3, training curriculum Multimedia Appendix 5, and incident reporting form Multimedia Appendix 7, also directly support institutional efforts in monitoring, training, and quality improvement.

These templates are intended as adaptable starting points, offering practical instruments that health care organizations and individuals can customize to their specific contexts and needs. Collectively, they aim to operationalize the principles of thoughtful use, support a culture of safety and continuous learning, and contribute to the responsible and ethical integration of LLMs in medicine.

### Strengths and Limitations

Strengths include (1) an interdisciplinary synthesis of AI safety, clinical ethics, and public-health harm reduction; (2) concrete, role-specific strategies linked to real-world scenarios; and (3) a forward-looking governance blueprint that acknowledges rapid model iteration. Limitations stem from the narrative rather than empirical design: effectiveness of proposed strategies is inferred from analogous interventions (eg, needle-exchange programs and clinical decision support validation studies) rather than prospectively tested; regulatory analysis is skewed toward US–European Union contexts; and emerging reasoning models [11] may render some recommendations particularly around knowledge cutoffs obsolete more quickly than anticipated.

### Future Directions

Rigorous prospective evaluations are needed to quantify how harm-reduction protocols affect diagnostic accuracy, workflow efficiency, clinician deskilling, and patient trust across diverse settings. Technical research should prioritize uncertainty quantification, bias-detection tooling, and privacy-preserving model fine-tuning on secure clinical data. Policy work must close liability gaps by clarifying the standard of care when LLMs contribute to clinical decisions. Finally, equity-focused studies should track whether LLM deployments narrow or widen digital-health disparities and test targeted interventions such as subsidized secure-access portals and culturally adapted health-literacy modules to ensure benefits accrue across demographic lines.

## Conclusions

LLMs offer significant potential in health care but carry inherent risks stemming from their technical limitations and the sensitive nature of medical information. Adopting a harm reduction framework acknowledges the inevitability of LLM use by patients and clinicians and prioritizes strategies to mitigate harm rather than futile attempts at prohibition. For patients, this involves fostering critical health literacy, promoting verification of LLM outputs against trusted sources, and safeguarding privacy through transparent data practices, turning passive information consumption into active, critical engagement. For clinicians, harm reduction necessitates rigorous training on LLM limitations and biases, enforcing mandatory “human-in-the-loop” validation for all clinical applications, and integrating LLMs strictly as assistive tools within secure, governed workflows, preventing diagnostic errors, deskilling, and bias propagation as illustrated by concrete examples.

Key challenges such as rapid technological evolution, regulatory ambiguity, bias amplification, implementation costs, and equity concerns demand collaborative, multi-stakeholder efforts among developers, institutions, policy makers, educators, and patients. Success hinges on balancing innovation with ethical imperatives: preserving nuanced clinical judgment, prioritizing patient safety above efficiency gains, and upholding trust in the patient-provider relationship. By embedding transparency, accountability, critical appraisal, and continuous oversight into LLM deployment, health care can harness the potential benefits of AI while actively safeguarding against its pitfalls. Ultimately, harm reduction is not a barrier to progress but a necessary, pragmatic pathway to ensure LLMs enhance, rather than undermine, the core values of equitable, evidence-based, and patient-centered medicine.

## Supplementary material

10.2196/75849 Multimedia Appendix 1 Using AI chat tools for health: a guide. AI: artificial intelligence.

10.2196/75849 Multimedia Appendix 2 Plain‑language AI glossary (patients). AI: artificial intelligence.

10.2196/75849 Multimedia Appendix 3 Structured evaluation log for clinical LLM prompts and versions. LLM: large language model.

10.2196/75849 Multimedia Appendix 4 Anonymous survey instrument: clinical LLM use and safety practices. LLM: large language model.

10.2196/75849 Multimedia Appendix 5 Foundational curriculum for clinician training on responsible and effective LLM use in health care. LLM: large language model.

10.2196/75849 Multimedia Appendix 6 Prompt library: clinician‑ready starters.

10.2196/75849 Multimedia Appendix 7 Standardized LLM-related clinical incident and near-miss reporting form. LLM: large language model.

10.2196/75849 Multimedia Appendix 8 Framework for institutional LLM governance committee: structure and mandate. LLM: large language model.

10.2196/75849 Multimedia Appendix 9 Safeguarding clinical acumen: strategies for maintaining and enhancing clinical reasoning skills in an LLM-integrated health care environment. LLM: large language model.

10.2196/75849 Multimedia Appendix 10 Summary of potential harms and examples of harm reduction strategies.

## Abbreviations

AI: artificial intelligence
GDPR: General Data Protection Regulation
HIPAA: Health Insurance Portability and Accountability Act
LLM: large language model
PHI: patient health information
