# Supplementary material for: Harm Reduction Strategies for Thoughtful Use of Large Language Models in the Medical Domain: Perspectives for Patients and Clinicians
Source: J Med Internet Res. 2025 Jul 25;27:e75849. doi: 10.2196/75849 (PMC12296254; doi:10.2196/75849)
Supplement: Multimedia Appendix 1 [file jmir-v27-e75849-s001.docx]

*This guide helps you get the most from large language model (LLM) chatbots—like ChatGPT or Gemini—while staying safe and informed.*

## **1. What’s an LLM, in one sentence?**

An LLM is a computer program that **predicts words** to answer questions or have a conversation; it is **not a doctor** and can be wrong.

## **2. Golden Rules**

1. **Treat answers as ideas, not prescriptions.** Always check with a health professional before acting.
2. **Look for the disclaimer.** If the chat tool shows a warning like “Not medical advice,” read it every time.
3. **Protect your privacy.** Never type your full name, date of birth, or medical record number.
4. **Cross‑check important facts.** Use trusted sites such as the CDC, WHO, or your local health authority.
5. **Ask, don’t diagnose.** Use prompts that start with *“Explain…”* or *“List resources about…”* rather than *“Do I have…”* or *“What pill should I take?”*

## **3. A Safe Chat in 4 Easy Steps**

| **Step** | **What you do** | **Why it helps** |
| --- | --- | --- |
| **1 Prepare** | Write down your symptoms or questions first. | Keeps the chat focused and clear. |
| **2 Prompt wisely** | Start with: *“Explain what happens in asthma in simple words.”* | You’ll get educational info, not a risky diagnosis. |
| **3 Verify** | Check two trusted sources (e.g., cdc.gov, cancer.org). | Confirms the answer is accurate. |
| **4 Follow‑up** | If worried, share what you found with your nurse or doctor. | Ensures a professional reviews any action. |

## **4. Smart Prompts to Try**

- “Explain iron‑deficiency anemia like I’m 12.”
- “List three reliable websites about managing type 2 diabetes.”
- “Give me questions I can ask my surgeon about knee replacement.”

**Avoid prompts like:** “Should I double my insulin dose now?”

## **5. Spotting Red Flags**

| Red flag | What to do |
| --- | --- |
| **The answer sounds certain but cites no source.** | Double‑check on a health‑authority website or call a clinic. |
| **You see medical words you don’t understand.** | Ask the bot: *“Define ____ in plain language.”* |
| **Advice conflicts with your doctor’s plan.** | Bring the chat text to your next appointment—do **not** change treatment on your own. |

## **6. Privacy Checklist**

- ☐ I removed my name and address.
- ☐ I did **not** upload scans, lab reports, or genetic data.
- ☐ I used a device I trust (not a public computer).
- ☐ I read the tool’s privacy page.

If any box is unchecked, fix it before you hit *Send*.

## **7. When to Stop Chatting and Call for Help**

Call your doctor, nurse line, or emergency services if you experience:

- Sudden chest pain, trouble breathing, or heavy bleeding.
- A new severe headache with fever or stiff neck.
- Suicidal thoughts or intent to harm yourself.

*AI chats can wait—your health can’t.*

## **8. Quick Resource List**

- **Find a doctor:** health.gov/find‑care
- **Reliable health info:** cdc.gov | who.int | medlineplus.gov
- **Mental‑health help (US):** 988 Suicide & Crisis Lifeline
