# Supplementary material for: Harm Reduction Strategies for Thoughtful Use of Large Language Models in the Medical Domain: Perspectives for Patients and Clinicians
Source: J Med Internet Res. 2025 Jul 25;27:e75849. doi: 10.2196/75849 (PMC12296254; doi:10.2196/75849)
Supplement: Multimedia Appendix 2 [file jmir-v27-e75849-s002.docx]

*25 key terms in ≤20 words with an everyday analogy*

| **Term** | **Plain definition** | **Everyday analogy** |
| --- | --- | --- |
| **1 Hallucination** | AI states untrue “facts.” | Like a friend confidently guessing an answer. |
| **2 Bias** | Systematic unfair tilt in outputs. | A loaded coin that lands heads more often. |
| **3 Bias audit** | Test to uncover those unfair tilts. | Checking many coin flips for imbalance. |
| **4 Temperature** | Setting that adds or reduces randomness. | Turning a spice dial up or down. |
| **5 Prompt** | The question or instruction you give. | A recipe request to a chef. |
| **6 Context window** | How much text the AI can “remember.” | A whiteboard that only fits so much. |
| **7 Token** | Small chunk of text the model reads. | Lego pieces building a sentence tower. |
| **8 Knowledge cutoff** | The newest date in the AI’s training. | A history book that ends in 2023. |
| **9 Model version** | Specific release of the AI. | Car model year (e.g., 2025, 2026). |
| **10 Human‑in‑the‑loop** | Required human review of AI output. | Pilot double‑checking autopilot settings. |
| **11 Retrieval‑Augmented Generation (RAG)** | AI adds fresh documents before replying. | Student grabs textbooks before answering. |
| **12 Embedding** | Numeric fingerprint of text meaning. | Latitude/longitude for sentences. |
| **13 Vector database** | Library that stores those fingerprints. | A map that finds nearby sentences. |
| **14 Parameter** | Learned weight inside the model. | Knob setting in a huge equalizer. |
| **15 Fine‑tuning** | Extra training for a niche task. | Teaching a violinist one new song. |
| **16 Guardrails** | Built‑in safety limits. | Speed bump that forces slower driving. |
| **17 Red teaming** | Stress‑testing for weaknesses. | Hiring hackers to break security. |
| **18 Prompt chaining** | Linking multiple prompts step‑by‑step. | Dominoes falling to reach a goal. |
| **19 Chain‑of‑thought** | AI shows its reasoning steps. | Showing long‑division work, not just answer. |
| **20 Reasoning model** | Latest AI that explains decisions. | Calculator that also shows formula. |
| **21 PHI (Protected Health Info)** | Identifiable personal medical data. | Name tag on a medical chart. |
| **22 Transparency report** | Public summary of how AI works. | Food label listing ingredients. |
| **23 Model card** | Snapshot of model limits & uses. | Car manual’s quick‑facts page. |
| **24 Redaction** | Removing sensitive text before use. | Black marker over secrets. |
| **25 Guarded output** | AI refuses risky request. | Locked door saying "Staff Only." |
