# Supplementary material for: Harm Reduction Strategies for Thoughtful Use of Large Language Models in the Medical Domain: Perspectives for Patients and Clinicians
Source: J Med Internet Res. 2025 Jul 25;27:e75849. doi: 10.2196/75849 (PMC12296254; doi:10.2196/75849)
Supplement: Multimedia Appendix 3 [file jmir-v27-e75849-s003.docx]

**Purpose.** This template standardizes how clinical staff document hands-on evaluations of large-language-model (LLM) prompts for specific clinical tasks. Completed logs populate a shared repository of **verified prompts**, each linked to the exact model build and annotated with safe-use guidance.
 **Confidentiality.** *Do not* enter protected health information (PHI). Logs are stored in the institution’s secure AI-governance archive.
 **How to use.** One log = one **model × prompt × clinical task**. Duplicate the blank table for every new evaluation and attach supporting artefacts (screenshots, outputs) if policy allows.

#### **Metadata**

| **Field** | **Entry** |
| --- | --- |
| Evaluator initials | ____ |
| Role / Specialty | ____ |
| Date (YYYY-MM-DD) | ____ |
| Clinical-task category | ☐ Documentation ☐ Decision-support ☐ Patient education ☐ Administrative ☐ Other → ____ |
| Patient-safety risk level † | ☐ Low ☐ Moderate ☐ High |
| Approved for production use? | ☐ Yes ☐ No ☐ Pending review |

† Use your institutional risk matrix (e.g., **High** = could directly influence diagnosis or treatment).

#### **1 | Model details**

| **Parameter** | **Entry** |
| --- | --- |
| Model name | ____ (e.g., *ChatGPT-4o*, *Med-PaLM 3*) |
| Exact version / build ID | ____ |
| Access environment | ☐ Public web ☐ Enterprise SaaS ☐ On-prem / local |
| Temperature / decoding settings | ____ |

#### **2 | Prompt & context**

| **Prompt label** | **____ (short title)** |
| --- | --- |
| **Full prompt text** | <paste here – no PHI> |
| System / developer message (if any) | ____ |
| Additional context injected (RAG, tools, etc.) | ____ |

#### **3 | Expected vs observed output**

| **Criterion** | **Expected behaviour** | **Observed outcome** | **Notes** |
| --- | --- | --- | --- |
| Accuracy / clinical correctness | ____ | ____ | ____ |
| Hallucination presence | “None” | ____ | ____ |
| Bias / equity issues | “None” | ____ | ____ |
| Readability & tone | ____ | ____ | ____ |
| Turn-around time (s) | ____ | ____ | ____ |

#### **4 | Verification steps taken**

- ☐ Cross-checked guideline / primary literature
- ☐ Second clinician review
- ☐ Secondary LLM consistency check
- ☐ PHI redaction confirmed
- Additional checks: ____

**Outcome of verification (pass/fail, required edits):** ____

#### **5 | Safety & deployment guidance**

1. **Recommended safe-use instructions** (e.g., “Only for draft generation; mandatory human sign-off before EHR entry”)
2. **Known failure modes / watch-outs**
3. **Suggested prompt refinements**

#### **6 | Overall evaluation rating (0–5)**

| **Scale** | **Definition** | **Score** |
| --- | --- | --- |
| 0 | Unacceptable – unsafe or useless | ☐0 |
| 1 | Poor – major issues | ☐1 |
| 2 | Fair – limited utility | ☐2 |
| 3 | Good – usable with vigilance | ☐3 |
| 4 | Very good – minor edits needed | ☐4 |
| 5 | Excellent – ready for production | ☐5 |

#### **7 | Review & approval *(to be completed by AI-safety lead)***

| **Field** | **Entry** |
| --- | --- |
| Reviewer initials | ____ |
| Review date | ____ |
| Decision | ☐ Approved ☐ Approved with conditions ☐ Rejected |
| Comments / conditions | ____ |
