# Supplementary material for: Harm Reduction Strategies for Thoughtful Use of Large Language Models in the Medical Domain: Perspectives for Patients and Clinicians
Source: J Med Internet Res. 2025 Jul 25;27:e75849. doi: 10.2196/75849 (PMC12296254; doi:10.2196/75849)
Supplement: Multimedia Appendix 4 [file jmir-v27-e75849-s004.docx]

**Purpose.** This confidential questionnaire gathers frontline insight into how health‑care staff actually employ large‑language‑model (LLM) tools. Results will feed a harm‑reduction programme where insights are used to improve education to limit harm and improve performance when using LLMs in the medical domain.

**Anonymity.** No names, e‑mail addresses, IP data or patient identifiers are collected. **Please omit all PHI** in free‑text fields.

**Time to complete.** ~ 7–10 minutes.

#### **Section S1.** Your clinical context.

1. **Primary role** (select one) ☐ Physician ☐ Nurse ☐ Pharmacist ☐ Allied‑health professional ☐ Administrator ☐ Other → ____
2. **Specialty / department** ____
3. **Years in practice** ☐ < 5 ☐ 5–10 ☐ 11–20 ☐ > 20
4. **Country/region of practice** ____

#### **Section S2.** LLMs you currently use.

*For each model you have used in the past three months, complete one row.*

| **LLM name (tick)** | **Exact version / build ID or date (free‑text)** | **Access environment (**☐ **Public web** ☐ **Enterprise/SaaS** ☐ **On‑prem/local)** |
| --- | --- | --- |
| ☐ ChatGPT | ____ | ____ |
| ☐ Gemini | ____ | ____ |
| ☐ Claude | ____ | ____ |
| ☐ Med‑PaLM | ____ | ____ |
| ☐ Locally‑hosted open‑source | ____ | ____ |
| ☐ Other → ____ | ____ | ____ |

#### **Section S3.** Typical tasks (tick all that apply).

☐ Drafting clinical notes ☐ Literature search / summarisation ☐ Patient‑education handouts ☐ Differential‑diagnosis brainstorming ☐ Coding / billing support ☐ Administrative e‑mails ☐ None of the above ☐ Other → ____

#### **Section S4.** Prompt engineering and examples.

1. **How often do you modify or engineer prompts to improve output quality?** ☐ Never ☐ Occasionally ☐ Often ☐ Always
2. **Please paste up to *three* prompts you consider most effective in your work.** (Do **not** include PHI.) • Prompt 1 ____ • Prompt 2 ____ • Prompt 3 ____
3. **Share one prompt that produced unsafe or low‑quality output and briefly explain what went wrong.**
4. **Do you share prompts with colleagues?** ☐ Never ☐ Occasionally ☐ Often If **yes**, where? ☐ Informal chat ☐ EHR "dot phrases" ☐ Central prompt library ☐ Other → ____

#### **Section S5.** Safety and verification practices.

1. **How often do you independently verify LLM output before clinical use?** ☐ Always ☐ > 75 % ☐ 25–75 % ☐ < 25 % ☐ Never
2. **Usual verification methods** (tick all) ☐ Check guideline / journal ☐ Cross‑reference EHR data ☐ Ask colleague ☐ Run second LLM ☐ No routine check
3. **Have you enabled any PHI‑redaction or pseudonymisation tools before sending data to an LLM?** ☐ Yes ☐ No ☐ Not sure
4. **List any additional safeguards you use (e.g., retrieval‑augmented generation, double‑reading, etc.)**

#### **Section S6.** Adverse events and near misses.

1. **Frequency of hallucinated or factually wrong output observed** ☐ Never ☐ 1–2 times ☐ 3–10 times ☐ > 10 times
2. **Did any erroneous output reach the patient record or influence care before detection?** ☐ Yes ☐ No
3. **Describe the most significant incident (no PHI). How was it detected and mitigated?**

#### **Section S7.** Institutional environment.

1. **Your organisation’s stance on LLM use** ☐ Officially approved ☐ Tolerated but unofficial ☐ Prohibited ☐ Unclear
2. **Available safeguards** (tick all) ☐ Secure, enterprise LLM platform ☐ Role‑based access ☐ Audit logs ☐ Mandatory training ☐ None / don’t know
3. **Has an external privacy / security audit been performed on the LLM environment in the past 12 months?** ☐ Yes ☐ No ☐ Don’t know

#### **Section S8.** Open‑ended reflections.

1. **Describe one scenario where an LLM *improved* patient care or workflow efficiency.**
2. **Describe one scenario where LLM use caused concern or potential harm.**
3. **What primary benefit motivates your LLM use?** (free‑text) ____
4. **What barrier most limits safer or broader LLM adoption in your setting?** (free‑text) ____
5. **If you could implement ONE policy or tool tomorrow to improve safe LLM use, what would it be and why?**
6. **Any additional comments, suggestions or resources worth sharing?**

#### **Section S9.** Follow‑up (optional).

Would you like to receive an aggregated summary of best‑practice tips? If yes, please provide a non‑work e‑mail via the separate, unlinkable form sent after submission.
