# Supplementary material for: Harm Reduction Strategies for Thoughtful Use of Large Language Models in the Medical Domain: Perspectives for Patients and Clinicians
Source: J Med Internet Res. 2025 Jul 25;27:e75849. doi: 10.2196/75849 (PMC12296254; doi:10.2196/75849)
Supplement: Multimedia Appendix 5 [file jmir-v27-e75849-s005.docx]

**Purpose:** This curriculum outline provides a foundational framework for healthcare institutions to develop comprehensive training programs for clinicians on the responsible, ethical, and effective use of Large Language Models (LLMs) in clinical practice. The goal is to equip clinicians with the necessary knowledge and skills to leverage LLM benefits while proactively mitigating potential harms, ensuring patient safety, and upholding medical professionalism. This training should be considered mandatory, role-specific where appropriate, and include competency assessments.

**Target Audience:** All clinical staff who may interact with or utilize LLM tools in their professional duties, including physicians, nurses, pharmacists, allied health professionals, and clinical administrators.

**Learning Objectives:** Upon completion of this training, clinicians should be able to:

1. Describe the fundamental principles, capabilities, and limitations of LLMs relevant to healthcare.
2. Identify and critically assess the potential risks and harms associated with LLM use in clinical settings, including inaccuracy, bias, privacy concerns, and deskilling.
3. Apply harm reduction strategies and "thoughtful use" protocols when interacting with LLMs for clinical tasks.
4. Effectively utilize institutionally approved LLM tools for sanctioned use cases while adhering to safety and verification protocols.
5. Recognize and begin to mitigate biases in LLM outputs.
6. Communicate transparently and appropriately with patients regarding the use of LLMs in their care, if applicable and as per institutional policy.
7. Understand their professional and ethical responsibilities, including accountability, when using LLMs.
8. Contribute to a culture of safety and continuous improvement regarding LLM use within their institution.

**Curriculum Modules:**

**Module 1: LLMs in the Medical Landscape – Fundamentals and Context**

- **1.1. Introduction to LLMs:**
  - What are LLMs? Simplified explanation of underlying technology (e.g., transformer models, training on vast datasets).
  - Key characteristics: Generative capabilities, natural language understanding, pattern recognition vs. true comprehension.
  - Distinction: General-purpose LLMs (e.g., public ChatGPT, Gemini) vs. medically fine-tuned, enterprise-grade models.
- **1.2. Current & Emerging Applications in Healthcare:**
  - Overview of LLM use cases (as outlined in the main paper, e.g., documentation, summarization, patient education drafting, literature review).
  - Potential benefits: Efficiency, decision support, enhanced communication (with caveats).
- **1.3. Institutional Framework for LLM Use:**
  - Overview of the institution's policies, guidelines, and governance structure for LLM use.
  - Approved LLM tools, platforms, and access protocols within the institution.
  - Explicitly prohibited uses (e.g., autonomous diagnosis, treatment decisions without human oversight).
  - The importance of using sanctioned tools vs. public, unsecured LLMs for clinical tasks.

**Module 2: Navigating the Risks – A Harm Reduction Perspective**

- **2.1. Accuracy, Misinformation, and Hallucinations:**
  - Understanding "hallucinations": Plausible-sounding but false information.
  - Knowledge cutoffs and outdated information.
  - Impact of inaccuracies on clinical decision-making and patient safety (referencing paper's Risk Matrix and use cases).
- **2.2. Bias and Equity:**
  - Sources of bias in LLMs (training data, algorithmic bias).
  - Manifestations: Racial, gender, socioeconomic, age, disability biases in outputs.
  - Potential for perpetuating health disparities and inequitable care.
- **2.3. Privacy, Data Security, and Regulatory Compliance:**
  - Risks associated with inputting Protected Health Information (PHI) into LLMs.
  - Compliance requirements (e.g., HIPAA, GDPR).
  - Institutional safeguards: Secure environments, data handling protocols for approved LLMs.
- **2.4. Cognitive and Professional Risks:**
  - Automation bias and over-reliance on LLM outputs.
  - Anchoring and premature closure in diagnostic reasoning.
  - Potential for deskilling in core clinical competencies.
  - The "black box" problem: Opacity of LLM decision-making processes.
  - Challenges with opaque model versioning and performance shifts.
- **2.5. Liability and Accountability:**
  - Current ambiguities in legal and professional responsibility.
  - Clinician accountability for all patient care decisions, even when assisted by LLMs.
  - Institutional policies on liability.

**Module 3: Core Principles for Thoughtful and Safe LLM Engagement**

- **3.1. The "Human-in-the-Loop" as a Non-Negotiable Standard:**
  - LLMs as assistive tools, not autonomous decision-makers.
  - The clinician's ultimate responsibility for reviewing, validating, and owning all clinical content and decisions.
- **3.2. Critical Appraisal and Verification of LLM Outputs:**
  - Developing a healthy skepticism: Questioning LLM-generated information.
  - Strategies for verification: Cross-referencing with evidence-based guidelines, primary literature, EHR data, expert consultation.
  - Recognizing context limitations of LLMs.
- **3.3. Safer Prompt Engineering for Clinical Utility:**
  - Techniques for crafting clear, specific, and unambiguous prompts.
  - Framing questions for informational/assistive purposes vs. diagnostic/prescriptive requests.
  - Understanding how prompt structure influences output quality and safety (linking to concepts in Appendix C).
  - Iterative prompting and refinement.
- **3.4. Managing Context Effectively and Safely:**
  - Awareness of context window limitations, especially in chat interfaces.
  - Risks of context degradation over long conversations.
  - Best practices for initiating new contexts for distinct tasks.

**Module 4: Practical Application and Workflow Integration**

- **4.1. Utilizing Approved LLM Tools within the Institution:**
  - Hands-on training with specific LLM platforms sanctioned by the institution.
  - Navigating security features, PHI handling, and integration with clinical workflows (e.g., EHR).
- **4.2. Active Bias Detection and Mitigation Strategies:**
  - Practical exercises in scrutinizing LLM outputs for subtle biases (e.g., language, assumptions, differential suggestions).
  - Techniques for editing and reframing LLM outputs to remove or mitigate bias.
  - Importance of diverse human review in identifying potential biases.
- **4.3. Implementing Safe Use Cases (Role-Specific Examples):**
  - **Documentation Assistance:** Drafting clinical notes, discharge summaries, referral letters (with mandatory review, editing, and validation protocols).
  - **Information Retrieval:** Assisting with literature searches, summarizing articles (with emphasis on source verification and critical appraisal of summaries).
  - **Patient Communication:** Drafting patient education materials, responses to patient queries (focusing on clarity, empathy, accuracy, and the need for personalization and professional review).
  - **Administrative Tasks:** Streamlining non-clinical communications, scheduling templates (while maintaining professionalism and accuracy).
- **4.4. Documenting LLM Use:**
  - Institutional policies on when and how to document the use of LLM assistance in patient records or clinical notes (if applicable).
  - Maintaining audit trails for accountability.

**Module 5: Ethical Imperatives and Patient-Centered Communication**

- **5.1. Transparency with Patients:**
  - Institutional guidelines on disclosing LLM use to patients.
  - When and how such disclosures should occur (e.g., for LLM-drafted educational materials).
  - Sample language and communication strategies for transparent and reassuring patient interactions.
- **5.2. Managing Patient-Initiated LLM Information:**
  - Approaches for constructively discussing health information patients bring from their own LLM interactions.
  - Guiding patients towards reliable resources and professional advice.
- **5.3. Upholding the Patient-Provider Relationship:**
  - Ensuring LLM use enhances, rather than depersonalizes, patient care.
  - Maintaining empathy, clinical judgment, and direct communication as primary.
- **5.4. Equity and Access in LLM-Assisted Care:**
  - Considering how LLM deployment might affect health equity.
  - Strategies to ensure equitable access to the benefits of LLM-assisted care and avoid widening disparities.

**Module 6: Governance, Continuous Learning, and Future Preparedness**

- **6.1. Reporting Incidents and Near Misses:**
  - Institutional procedures for reporting errors, biases, or safety concerns related to LLM use.
  - Contributing to a learning health system approach for LLM deployment.
- **6.2. Role in Institutional LLM Governance:**
  - Understanding how clinicians can provide feedback (e.g., through surveys like Appendix B, evaluation logs like Appendix C).
  - Participating in ongoing review and refinement of LLM use policies and practices.
- **6.3. Staying Abreast of LLM Evolution:**
  - Recognizing the rapid pace of LLM development.
  - Commitment to ongoing professional development in AI and digital health.
  - Resources for staying updated on new LLM capabilities, risks, and best practices.
- **6.4. Adapting to Evolving Regulatory and Ethical Landscapes.**

**Assessment and Competency:**

- Pre- and post-training knowledge assessments.
- Scenario-based exercises (e.g., evaluating LLM outputs for specific tasks, identifying biases, formulating safe prompts).
- Observed structured clinical exercises (OSCEs) for select roles or tasks involving LLM interaction.
- Certification or documentation of completed training and demonstrated competency, as per institutional policy.
- Requirement for periodic refresher training and competency checks.

**Appendix F: Safeguarding Clinical Acumen: Strategies for Maintaining and Enhancing Clinical Reasoning Skills in an LLM-Integrated Healthcare Environment**

**Purpose:** The integration of Large Language Models (LLMs) into healthcare offers significant assistive potential. However, a critical long-term concern is the potential for "deskilling"—the erosion of core clinical reasoning and diagnostic competencies among clinicians who may become overly reliant on these tools for cognitive tasks. This appendix outlines proactive strategies for individual clinicians, medical educators, and healthcare institutions to safeguard and actively cultivate these essential human skills in an environment increasingly augmented by AI. Its aim is to ensure that LLMs serve as tools that augment, rather than diminish, the profound expertise of healthcare professionals.

**1. Understanding the Risk: LLM-Associated Deskilling**

- **Cognitive Offloading:** LLMs can efficiently perform tasks like information synthesis, differential diagnosis generation, and note drafting. While beneficial for reducing burden, routine offloading of these cognitive processes can reduce opportunities for clinicians to practice and refine their own reasoning skills.
- **Automation Bias & Premature Closure:** Over-reliance can lead to uncritical acceptance of LLM outputs (automation bias) or premature narrowing of diagnostic possibilities, hindering thorough analytical thinking.
- **Erosion of Foundational Knowledge Recall:** Reduced need to actively recall and apply foundational medical knowledge for certain tasks might weaken long-term retention and integration.
- **Impact on Novices vs. Experts:** While experts might use LLMs to confirm or expand their thinking, novices might be more susceptible to relying on LLMs as a primary reasoning engine, potentially stunting the development of their own clinical judgment.

**2. Strategies for Individual Clinicians**

- **Mindful Metacognition and Self-Correction:**
  - **"Human First" Principle:** Before consulting an LLM for complex tasks (e.g., differential diagnosis, treatment planning), consciously formulate your own thoughts, hypotheses, and plans first. Use the LLM output as a comparator or for augmentation, not as a starting point.
  - **Articulate Your Reasoning:** Verbally or mentally justify your clinical decisions independently before comparing with LLM suggestions. Note discrepancies and critically analyze why they occurred.
  - **Seek Disconfirming Evidence:** Actively challenge both your own initial conclusions and those suggested by the LLM.
- **Deliberate Practice and Skill Reinforcement:**
  - **Periodic "LLM-Free" Work:** Intentionally complete certain tasks or case reviews without LLM assistance to reinforce your own cognitive pathways.
  - **Engage with Complexity:** Actively seek out and manage complex cases that require deep reasoning, rather than deferring primarily to AI for these challenges.
  - **Teach and Mentor:** Explaining clinical reasoning to students, residents, or colleagues is a powerful way to solidify one's own understanding and identify gaps.
- **Critical and Reflective Engagement with LLMs:**
  - **Treat LLMs as "Consultants with Limitations":** Approach LLM outputs with the same critical appraisal you would apply to information from any other source, being acutely aware of their potential for error and bias.
  - **Analyze LLM "Reasoning" (If Available):** For models that provide reasoning traces, scrutinize these critically. Don't just look at the answer; assess the pathway.
  - **Focus on Learning:** Use LLMs as tools to explore knowledge gaps identified in your own reasoning, prompting them for explanations of concepts or alternative perspectives once you've established your own baseline.

**3. Adaptations for Medical Education and Training Programs**

- **Curriculum Integration:**
  - **Teach Clinical Reasoning Explicitly:** Reinforce foundational principles of diagnostic reasoning, clinical decision-making, and managing uncertainty *before* introducing LLM tools.
  - **AI Literacy with a Critical Lens:** Educate trainees on LLM capabilities and limitations, specifically focusing on risks like automation bias and deskilling (referencing concepts from Appendix E).
  - **Comparative Reasoning Exercises:** Design learning activities where trainees first develop their own diagnostic or management plans, then compare and critique them against LLM-generated options, justifying their final reconciled plan.
- **Pedagogical Approaches:**
  - **Simulation-Based Learning:** Use simulations to allow trainees to practice decision-making with and without LLM assistance, followed by debriefing sessions focused on reasoning processes and appropriate AI use.
  - **Problem-Based Learning (PBL) with AI Context:** Adapt PBL cases to include scenarios where LLM information (potentially flawed or biased) is introduced, requiring students to critically evaluate and integrate it.
  - **Mentorship in the Age of AI:** Encourage faculty to explicitly discuss and model how they balance their own expertise with the use of AI tools, emphasizing lifelong learning and skill maintenance.
- **Assessment Strategies:**
  - Assess clinical reasoning skills independently of LLM use.
  - Develop assessments that evaluate a trainee's ability to *critically appraise and appropriately integrate* LLM-generated information, rather than just the correctness of the final answer.

**4. Institutional Support and Cultural Reinforcement**

- **Promote a Culture of Intellectual Humility and Critical Thinking:**
  - Encourage open discussion about the limitations of AI and the irreplaceability of human clinical judgment.
  - Foster an environment where questioning LLM outputs is valued.
- **Provide Resources and Protected Time:**
  - Support continuous professional development focused on advanced clinical reasoning and critical appraisal skills.
  - Consider protected time for activities that promote deep thinking and skill maintenance, such as grand rounds focused on complex diagnostic challenges without initial AI input, or peer case discussions.
- **Thoughtful LLM Implementation:**
  - Deploy LLMs primarily as tools to *augment* human expertise and reduce administrative burden, rather than as replacements for core clinical cognitive functions.
  - Design workflows that reinforce the "human-in-the-loop" principle and provide clear checkpoints for human validation and judgment.
- **Monitor and Evaluate:**
  - Consider methods (e.g., surveys, audits of reasoning in complex cases) to periodically assess the potential impact of LLM integration on clinician skills and adapt strategies as needed.
  - Solicit clinician feedback on how LLM tools are affecting their practice and cognitive load.

**Conclusion:** The integration of LLMs into healthcare is not merely a technological shift but a professional one. By proactively adopting strategies at the individual, educational, and institutional levels, the healthcare community can harness the power of LLMs to enhance care while simultaneously safeguarding and cultivating the invaluable clinical acumen that lies at the heart of excellent patient care. Continuous vigilance and adaptation will be key to navigating this evolving landscape successfully.

# **Appendix G – Plain‑Language AI Glossary (Patients)**

*25 key terms in ≤20 words with an everyday analogy*

| **Term** | **Plain definition** | **Everyday analogy** |
| --- | --- | --- |
| **1 Hallucination** | AI states untrue “facts.” | Like a friend confidently guessing an answer. |
| **2 Bias** | Systematic unfair tilt in outputs. | A loaded coin that lands heads more often. |
| **3 Bias audit** | Test to uncover those unfair tilts. | Checking many coin flips for imbalance. |
| **4 Temperature** | Setting that adds or reduces randomness. | Turning a spice dial up or down. |
| **5 Prompt** | The question or instruction you give. | A recipe request to a chef. |
| **6 Context window** | How much text the AI can “remember.” | A whiteboard that only fits so much. |
| **7 Token** | Small chunk of text the model reads. | Lego pieces building a sentence tower. |
| **8 Knowledge cutoff** | The newest date in the AI’s training. | A history book that ends in 2023. |
| **9 Model version** | Specific release of the AI. | Car model year (e.g., 2025, 2026). |
| **10 Human‑in‑the‑loop** | Required human review of AI output. | Pilot double‑checking autopilot settings. |
| **11 Retrieval‑Augmented Generation (RAG)** | AI adds fresh documents before replying. | Student grabs textbooks before answering. |
| **12 Embedding** | Numeric fingerprint of text meaning. | Latitude/longitude for sentences. |
| **13 Vector database** | Library that stores those fingerprints. | A map that finds nearby sentences. |
| **14 Parameter** | Learned weight inside the model. | Knob setting in a huge equalizer. |
| **15 Fine‑tuning** | Extra training for a niche task. | Teaching a violinist one new song. |
| **16 Guardrails** | Built‑in safety limits. | Speed bump that forces slower driving. |
| **17 Red teaming** | Stress‑testing for weaknesses. | Hiring hackers to break security. |
| **18 Prompt chaining** | Linking multiple prompts step‑by‑step. | Dominoes falling to reach a goal. |
| **19 Chain‑of‑thought** | AI shows its reasoning steps. | Showing long‑division work, not just answer. |
| **20 Reasoning model** | Latest AI that explains decisions. | Calculator that also shows formula. |
| **21 PHI (Protected Health Info)** | Identifiable personal medical data. | Name tag on a medical chart. |
| **22 Transparency report** | Public summary of how AI works. | Food label listing ingredients. |
| **23 Model card** | Snapshot of model limits & uses. | Car manual’s quick‑facts page. |
| **24 Redaction** | Removing sensitive text before use. | Black marker over secrets. |
| **25 Guarded output** | AI refuses risky request. | Locked door saying "Staff Only." |
