# Supplementary material for: Harm Reduction Strategies for Thoughtful Use of Large Language Models in the Medical Domain: Perspectives for Patients and Clinicians
Source: J Med Internet Res. 2025 Jul 25;27:e75849. doi: 10.2196/75849 (PMC12296254; doi:10.2196/75849)
Supplement: Multimedia Appendix 6 [file jmir-v27-e75849-s006.docx]

*20 task‑tagged templates with risk level*

The risk level is an assessment of the relative risk of a query. Risk assessments **does not** qualify as institutional approval to use LLMs for a task.

**Legend

Risk**Low (L) – no clinical decision;
Moderate (M) – may influence care;
High (H) – diagnostic / treatment impact.

| # | Task Tag | Prompt Starter (copy ➜ paste variables in {{braces}}) | Risk |
| --- | --- | --- | --- |
| 1 | **Doc‑Summary** | "Summarise this encounter note into a problem‑oriented SOAP. Use bullet Points.\n\nNote:\n{{CLINICAL_NOTE}}" | M |
| 2 | **Discharge‑Letter** | "Draft a patient‑friendly discharge letter at 8th‑grade reading level. Key points: {{KEY_POINTS}}" | M |
| 3 | **Referral‑Letter** | "Compose a referral to {{SPECIALTY}} including salient history, exam, and pending tests from this synopsis: {{SUMMARY}}" | M |
| 4 | **Lab‑Explain** | "Explain these lab results in plain English for a patient: {{LAB_TABLE}}" | L |
| 5 | **Patient‑Question‑Prep** | "List 5 questions a patient with {{CONDITION}} should ask at their next visit." | L |
| 6 | **Differential diagnostics** | "Given age {{AGE}}, sex {{SEX}}, and symptoms {{SYMPTOMS}}, list a differential (max 10) ranked by likelihood." | H |
| 7 | **Imaging‑Justify** | "For suspected {{CONDITION}}, summarise guideline‑based indications for ordering {{IMAGING}}." | M |
| 8 | **Guideline‑Compare** | "Contrast 2023 vs 2025 guidelines for {{TOPIC}} in 150 words." | L |
| 9 | **Lit‑Search** | "Return 5 high‑quality RCTs on {{QUESTION}} | M |
| 10 | **Drug‑Info** | "Summarise mechanism, dosing, and main serious ADRs of {{DRUG}}." | M |
| 11 | **Patient‑Handout** | "Create a 250‑word handout on {{CONDITION}} including lifestyle tips, at 8th‑grade level." | L |
| 12 | **Coding‑Check** | "Suggest the most specific ICD‑10 code(s) for this summary: {{SUMMARY}}." | M |
| 13 | **Admin‑Email** | "Draft a polite email to schedule {{ACTION}} with {{STAKEHOLDER}}. Tone: concise, professional." | L |
| 14 | **Policy‑Digest** | "Bullet‑point the key changes in the latest hospital policy on {{TOPIC}} (max 12 bullets)." | L |
| 15 | **Consult‑Prep** | "Generate focused questions to ask {{SPECIALTY}} regarding {{PATIENT_ISSUE}}." | M |
| 16 | **Risk‑Explain** | "Explain absolute vs relative risk using these statistics: {{DATA}} – keep below 150 words." | L |
| 17 | **Bias‑Scan** | "Review this draft note for language bias related to race, gender, age. Suggest neutral rewrites." | M |
| 18 | **Second‑Opinion** | "List possible alternate diagnoses not yet considered for case: {{CASE_SYNOPSIS}}." | H |
