# Supplementary material for: Harm Reduction Strategies for Thoughtful Use of Large Language Models in the Medical Domain: Perspectives for Patients and Clinicians
Source: J Med Internet Res. 2025 Jul 25;27:e75849. doi: 10.2196/75849 (PMC12296254; doi:10.2196/75849)
Supplement: Multimedia Appendix 7 [file jmir-v27-e75849-s007.docx]

**Purpose:** To confidentially report and analyze clinical incidents or near-misses involving Large Language Models (LLMs) to improve safety and system performance. This reporting process is vital for continuous learning and upholding patient safety.

**Confidentiality & Non-Punitive Reporting:** Information provided on this form will be treated confidentially. This reporting system is designed to be non-punitive and focuses on system improvement.

**Section S1.** Event details.

1.1. **Date of Event/Discovery:** ____ / ____ / ________ (DD/MM/YYYY)

1.2. **Time of Event/Discovery (approx.):** ____ : ____ (HH:MM, 24-hour format)

**Section S2.** Reporter information (optional).

2.1. **Reporter Role/Position:** ______________________________________ *(Examples: Physician, Nurse, Pharmacist, Clinical Coder, Administrative Staff, etc.)*

2.2. **Department/Unit:** ______________________________________

2.3. **Reporter ID (if anonymized system used):** _________________________

**Section S3.** LLM and task details.

3.1. **LLM Tool Used:** * Name: ______________________________________ * Version (if known/applicable): ______________________________________ *(e.g., ChatGPT-4o, Gemini Advanced, Institution-Specific Model v1.2)*

3.2. **Clinical Task Being Performed with LLM:** ________________________________________________________________________________ ________________________________________________________________________________ *(Examples: Drafting patient summary, generating differential diagnosis list, literature search, transcribing notes, patient communication draft, etc.)*

**Section 4.** Description of event and near-miss.

4.1. **Detailed Description of What Happened:** *(Please be specific. What were you trying to do? What steps did you take?)* ________________________________________________________________________________ ________________________________________________________________________________ ________________________________________________________________________________ ________________________________________________________________________________

4.2. **LLM Output (if applicable and can be safely recalled/copied without PHI):** *(Or describe the nature of the output if exact wording cannot be provided.)* ________________________________________________________________________________ ________________________________________________________________________________ ________________________________________________________________________________

4.3. **What was the Expected/Desired Outcome or LLM Output?** ________________________________________________________________________________ ________________________________________________________________________________ ________________________________________________________________________________

**Section S5.** Impact assessment.

5.1. **Patient Impact (select one):**
* [ ] **None:** No discernible impact on the patient.
* [ ] **Minor:** e.g., slight delay in care, patient confusion resolved quickly, minor inconvenience.
* [ ] **Moderate:** e.g., required additional intervention/consultation, significant delay, emotional distress.

* [ ] **Major:** e.g., resulted in significant harm, life-threatening situation, incorrect treatment initiated (even if caught).
* [ ] **Near-Miss:** An error occurred that had the potential to cause harm but did not reach the patient or cause harm due to timely intervention or chance.
* [ ] **Uncertain/Unknown.**

5.2. **De-identified Context of Patient Impact (if any, and "None" not selected):** *(DO NOT include any Patient Identifiable Information.)* ________________________________________________________________________________ ________________________________________________________________________________

**Section S6.** Detection of issue.

6.1. **How was the issue/error detected? (select primary method):** * [ ] Clinician's own review/verification prior to action. * [ ] Review by another clinical staff member. * [ ] Patient feedback or questioning. * [ ] Automated system alert/flag (if applicable). * [ ] During a quality assurance review. * [ ] Other (please specify): ______________________________________

**Section S7.** Perceived contributing factors (select all that apply).

- [ ] **LLM Inaccuracy/Hallucination:** (Output factually incorrect or fabricated).
- [ ] **LLM Bias:** (Output reflected demographic, social, or other bias).
- [ ] **User Error/Misinterpretation:** (e.g., incorrect prompt, misunderstanding of output).
- [ ] **Workflow Integration Issue:** (e.g., problem with how LLM fits into clinical process).
- [ ] **Technical Glitch/System Error:** (e.g., LLM platform malfunctioned).
- [ ] **LLM Knowledge Cutoff/Outdated Information.**
- [ ] **Lack of LLM Training/Familiarity.**
- [ ] **Time Pressure/High Workload.**
- [ ] **Unclear Institutional Guidelines for LLM Use.**
- [ ] **Other (please specify):** ________________________________________________________________________________ ________________________________________________________________________________

**Section S8.** Actions and prevention.

8.1. **Actions Taken Immediately (to mitigate risk or correct the issue):** ________________________________________________________________________________ ________________________________________________________________________________ ________________________________________________________________________________

8.2. **Suggestions for Prevention (What could prevent this from happening again?):** *(Consider changes to technology, workflow, training, policy, etc.)* ________________________________________________________________________________ ________________________________________________________________________________ ________________________________________________________________________________

**Reporting Protocol:**

- **Submission:** Please submit this completed form to the designated office/individual (e.g., Patient Safety Officer, LLM Governance Committee Secretariat, Quality Improvement Department) as per institutional guidelines.
- **Review:** Reports will be reviewed in a timely manner to identify trends, learn from events, and implement system improvements.
- **Feedback:** Aggregated, de-identified findings and implemented changes may be communicated back to staff through established channels to promote shared learning.
