# Supplementary material for: Harm Reduction Strategies for Thoughtful Use of Large Language Models in the Medical Domain: Perspectives for Patients and Clinicians
Source: J Med Internet Res. 2025 Jul 25;27:e75849. doi: 10.2196/75849 (PMC12296254; doi:10.2196/75849)
Supplement: Multimedia Appendix 8 [file jmir-v27-e75849-s008.docx]

The rapid integration of Large Language Models (LLMs) into healthcare presents significant opportunities alongside considerable risks. Effective governance is paramount to harness the benefits of LLMs while mitigating potential harms to patients, clinicians, and the institution. This framework provides a template for establishing an LLM Governance Committee (LGC), an interdisciplinary body responsible for overseeing the safe, ethical, and effective deployment and use of LLMs within the healthcare organization. This document outlines the LGC's purpose, mandate, composition, operational structure, and key performance indicators, drawing upon the principles of continuous interdisciplinary oversight.

**1. Purpose of the LLM Governance Committee (LGC)**

The primary purpose of the LGC is to provide strategic oversight and expert guidance for all aspects of LLM utilization within the institution. This includes ensuring that LLM deployment and use align with organizational values, ethical principles, regulatory requirements, patient safety standards, and clinical best practices, thereby fostering responsible innovation.

**2. Mandate and Key Responsibilities**

The LGC shall have the authority and responsibility to:

- **2.1. Policy Development and Oversight:**
  - Develop, implement, and regularly review institutional policies, procedures, and guidelines for the procurement, deployment, and use of LLMs in clinical and administrative settings.
  - Ensure policies address data privacy, security, patient consent, approved use cases, and prohibited activities.
- **2.2. Risk Assessment and Management for New LLM Tools/Uses:**
  - Establish and oversee a standardized process for evaluating and approving new LLM applications or significant updates to existing ones.
  - Conduct comprehensive risk assessments, considering clinical safety, bias, equity, data security, and operational impact before deployment.
  - Define risk mitigation strategies and ensure their implementation.
- **2.3. Incident Review and Organizational Learning:**
  - Establish and oversee a system for reporting, investigating, and analyzing LLM-related incidents, near-misses, and adverse events.
  - Identify root causes and systemic vulnerabilities, and disseminate lessons learned to prevent recurrence.
  - Recommend corrective and preventive actions.
- **2.4. Ethical Guidance and Oversight:**
  - Provide guidance on ethical dilemmas arising from LLM use in healthcare.
  - Ensure LLM applications respect patient autonomy, promote equity, and maintain trust in the patient-provider relationship.
  - Address issues of bias in LLM algorithms and outputs and promote strategies for their detection and mitigation.
- **2.5. Education and Training Strategy (Clinician and Patient):**
  - Oversee the development and implementation of comprehensive LLM training programs for clinicians and relevant staff, covering capabilities, limitations, ethical use, and institutional policies.
  - Guide strategies for educating patients about how LLMs may be used in their care and how their data is handled..
- **2.6. Performance Monitoring and Impact Assessment:**
  - Define metrics and processes for monitoring the performance, safety, and impact of LLMs used within the institution.
  - Regularly assess the benefits and drawbacks of LLM integration on clinical workflows, clinician well-being including deskilling concerns, patient outcomes, and health equity.
- **2.7. Stakeholder Engagement and Communication:**
  - Facilitate communication and collaboration between clinical staff, IT, legal, ethics, patients, and other stakeholders regarding LLM governance.
  - Ensure transparency regarding the institution's LLM strategy and governance processes.

**3. Proposed Interdisciplinary Composition**

The LGC should comprise representatives with diverse expertise to ensure comprehensive perspectives. Membership should include, but not be limited to:

- **Clinical Leadership:** Physicians from various key specialties (e.g., medicine, surgery, radiology, pathology), ensuring broad clinical input.
- **Nursing Leadership:** Representatives from nursing management and frontline nursing staff.
- **Information Technology (IT) / Data Security Leadership:** Chief Information Officer (CIO), Chief Information Security Officer (CISO), or their designees, responsible for technical infrastructure, data management, and cybersecurity.
- **Legal Counsel / Compliance Officer:** To advise on regulatory requirements (e.g., HIPAA, GDPR), liability, and institutional risk.
- **Clinical Ethicist:** To provide expertise on ethical principles and guide decision-making.
- **Patient Representatives / Advocates:** To ensure patient perspectives, values, and concerns are central to governance decisions.
- **Data Scientists / AI Specialists:** Individuals with technical expertise in AI/LLMs (may be internal staff, consultants, or academic partners).
- **Quality Improvement / Patient Safety Officer(s):** To integrate LLM safety with broader patient safety initiatives and quality frameworks.
- **Health Informatics Leadership:** Experts in clinical information systems, data standards, and workflow integration.
- **Administrative/Operational Leadership:** Representatives who can speak to resource allocation, operational feasibility, and change management.

**4. Operational Structure**

- **4.1. Chairperson:**
  - The LGC should be chaired by a respected senior leader within the institution (e.g., Chief Medical Officer, Chief Quality Officer) with the authority to champion its recommendations.
  - The Chairperson is responsible for leading meetings, setting agendas, and ensuring the committee fulfills its mandate.
- **4.2. Reporting Lines:**
  - The LGC should report to a high-level institutional body, such as the Executive Leadership Team, Medical Executive Committee, or Board of Trustees, to ensure accountability and facilitate the implementation of its recommendations.
- **4.3. Meeting Frequency:**
  - Regular meetings are essential. A suggested cadence is monthly during initial setup and active LLM rollouts, transitioning to quarterly once stable processes are established. Ad-hoc meetings may be called as needed to address urgent issues.
- **4.4. Quorum and Decision-Making:**
  - Define quorum requirements for meetings.
  - Decisions should ideally be made by consensus. A clear process for voting or escalating unresolved issues should be established if consensus cannot be reached.
- **4.5. Subcommittee Structure (Optional but Recommended):**
  - The LGC may establish standing or ad-hoc subcommittees to manage specific tasks, for example:
    - *Technical Evaluation Subcommittee:* Reviews technical aspects, security, and integration of new LLM tools.
    - *Ethical Review Subcommittee:* Provides in-depth analysis of ethical implications for specific use cases.
    - *Policy Review Subcommittee:* Drafts and revises LLM-related policies.
    - *Incident Analysis Subcommittee:* Reviews reported LLM-related incidents and proposes actions.
  - Subcommittees would report their findings and recommendations to the full LGC.
- **4.6. Documentation and Record Keeping:**
  - Formal minutes of all LGC meetings, including decisions made, action items, and responsible parties, must be maintained.
  - A central repository for all LLM-related policies, risk assessments, and governance documents should be established and kept current.
- **4.7. Resource Allocation:**
  - The institution must provide adequate administrative support, and potentially dedicated staff time or technical resources, to enable the LGC to function effectively.

**5. Key Performance Indicators (KPIs) for the LGC**

The effectiveness of the LGC can be monitored through various KPIs, including:

- Number of LLM-related policies and guidelines developed, reviewed, and updated annually.
- Average turnaround time for conducting risk assessments and providing recommendations for new LLM tools or use cases.
- Number of LLM-related incidents reported, reviewed, and percentage of those with actionable recommendations implemented within a defined timeframe.
- Clinician engagement levels (e.g., participation in training, utilization of approved tools, feedback on LLM initiatives).
- Results from clinician satisfaction surveys regarding LLM tools, support, and training.
- Patient feedback (e.g., through surveys or focus groups) regarding transparency and understanding of LLM use in their care.
- Rates of adherence to institutional LLM usage policies, identified through audits or other monitoring mechanisms.
- Evidence of bias mitigation efforts and monitoring for equitable LLM performance.

**6. Review and Adaptation**

The LGC's structure, mandate, composition, and operational procedures should be reviewed periodically (e.g., annually or biennially) and adapted as needed to respond to the evolving landscape of LLM technology, regulatory changes, institutional priorities, and lessons learned from its own operations.
