# Supplementary material for: Harm Reduction Strategies for Thoughtful Use of Large Language Models in the Medical Domain: Perspectives for Patients and Clinicians
Source: J Med Internet Res. 2025 Jul 25;27:e75849. doi: 10.2196/75849 (PMC12296254; doi:10.2196/75849)
Supplement: Multimedia Appendix 9 [file jmir-v27-e75849-s009.docx]

**Purpose:** The integration of Large Language Models (LLMs) into healthcare offers significant assistive potential. However, a critical long-term concern is the potential for "deskilling"—the erosion of core clinical reasoning and diagnostic competencies among clinicians who may become overly reliant on these tools for cognitive tasks. This appendix outlines proactive strategies for individual clinicians, medical educators, and healthcare institutions to safeguard and actively cultivate these essential human skills in an environment increasingly augmented by AI. Its aim is to ensure that LLMs serve as tools that augment, rather than diminish, the profound expertise of healthcare professionals.

**1. Understanding the Risk: LLM-Associated Deskilling**

- **Cognitive Offloading:** LLMs can efficiently perform tasks like information synthesis, differential diagnosis generation, and note drafting. While beneficial for reducing burden, routine offloading of these cognitive processes can reduce opportunities for clinicians to practice and refine their own reasoning skills.
- **Automation Bias & Premature Closure:** Over-reliance can lead to uncritical acceptance of LLM outputs (automation bias) or premature narrowing of diagnostic possibilities, hindering thorough analytical thinking.
- **Erosion of Foundational Knowledge Recall:** Reduced need to actively recall and apply foundational medical knowledge for certain tasks might weaken long-term retention and integration.
- **Impact on Novices vs. Experts:** While experts might use LLMs to confirm or expand their thinking, novices might be more susceptible to relying on LLMs as a primary reasoning engine, potentially stunting the development of their own clinical judgment.

**2. Strategies for Individual Clinicians**

- **Mindful Metacognition and Self-Correction:**
  - **"Human First" Principle:** Before consulting an LLM for complex tasks (e.g., differential diagnosis, treatment planning), consciously formulate your own thoughts, hypotheses, and plans first. Use the LLM output as a comparator or for augmentation, not as a starting point.
  - **Articulate Your Reasoning:** Verbally or mentally justify your clinical decisions independently before comparing with LLM suggestions. Note discrepancies and critically analyze why they occurred.
  - **Seek Disconfirming Evidence:** Actively challenge both your own initial conclusions and those suggested by the LLM.
- **Deliberate Practice and Skill Reinforcement:**
  - **Periodic "LLM-Free" Work:** Intentionally complete certain tasks or case reviews without LLM assistance to reinforce your own cognitive pathways.
  - **Engage with Complexity:** Actively seek out and manage complex cases that require deep reasoning, rather than deferring primarily to AI for these challenges.
  - **Teach and Mentor:** Explaining clinical reasoning to students, residents, or colleagues is a powerful way to solidify one's own understanding and identify gaps.
- **Critical and Reflective Engagement with LLMs:**
  - **Treat LLMs as "Consultants with Limitations":** Approach LLM outputs with the same critical appraisal you would apply to information from any other source, being acutely aware of their potential for error and bias.
  - **Analyze LLM "Reasoning" (If Available):** For models that provide reasoning traces, scrutinize these critically. Don't just look at the answer; assess the pathway.
  - **Focus on Learning:** Use LLMs as tools to explore knowledge gaps identified in your own reasoning, prompting them for explanations of concepts or alternative perspectives once you've established your own baseline.

**3. Adaptations for Medical Education and Training Programs**

- **Curriculum Integration:**
  - **Teach Clinical Reasoning Explicitly:** Reinforce foundational principles of diagnostic reasoning, clinical decision-making, and managing uncertainty *before* introducing LLM tools.
  - **AI Literacy with a Critical Lens:** Educate trainees on LLM capabilities and limitations, specifically focusing on risks like automation bias and deskilling (referencing concepts from Appendix E).
  - **Comparative Reasoning Exercises:** Design learning activities where trainees first develop their own diagnostic or management plans, then compare and critique them against LLM-generated options, justifying their final reconciled plan.
- **Pedagogical Approaches:**
  - **Simulation-Based Learning:** Use simulations to allow trainees to practice decision-making with and without LLM assistance, followed by debriefing sessions focused on reasoning processes and appropriate AI use.
  - **Problem-Based Learning (PBL) with AI Context:** Adapt PBL cases to include scenarios where LLM information (potentially flawed or biased) is introduced, requiring students to critically evaluate and integrate it.
  - **Mentorship in the Age of AI:** Encourage faculty to explicitly discuss and model how they balance their own expertise with the use of AI tools, emphasizing lifelong learning and skill maintenance.
- **Assessment Strategies:**
  - Assess clinical reasoning skills independently of LLM use.
  - Develop assessments that evaluate a trainee's ability to *critically appraise and appropriately integrate* LLM-generated information, rather than just the correctness of the final answer.

**4. Institutional Support and Cultural Reinforcement**

- **Promote a Culture of Intellectual Humility and Critical Thinking:**
  - Encourage open discussion about the limitations of AI and the irreplaceability of human clinical judgment.
  - Foster an environment where questioning LLM outputs is valued.
- **Provide Resources and Protected Time:**
  - Support continuous professional development focused on advanced clinical reasoning and critical appraisal skills.
  - Consider protected time for activities that promote deep thinking and skill maintenance, such as grand rounds focused on complex diagnostic challenges without initial AI input, or peer case discussions.
- **Thoughtful LLM Implementation:**
  - Deploy LLMs primarily as tools to *augment* human expertise and reduce administrative burden, rather than as replacements for core clinical cognitive functions.
  - Design workflows that reinforce the "human-in-the-loop" principle and provide clear checkpoints for human validation and judgment.
- **Monitor and Evaluate:**
  - Consider methods (e.g., surveys, audits of reasoning in complex cases) to periodically assess the potential impact of LLM integration on clinician skills and adapt strategies as needed.
  - Solicit clinician feedback on how LLM tools are affecting their practice and cognitive load.

**Conclusion:** The integration of LLMs into healthcare is not merely a technological shift but a professional one. By proactively adopting strategies at the individual, educational, and institutional levels, the healthcare community can harness the power of LLMs to enhance care while simultaneously safeguarding and cultivating the invaluable clinical acumen that lies at the heart of excellent patient care. Continuous vigilance and adaptation will be key to navigating this evolving landscape successfully.
