# Supplementary material for: Harm Reduction Strategies for Thoughtful Use of Large Language Models in the Medical Domain: Perspectives for Patients and Clinicians
Source: J Med Internet Res. 2025 Jul 25;27:e75849. doi: 10.2196/75849 (PMC12296254; doi:10.2196/75849)
Supplement: Multimedia Appendix 10 [file jmir-v27-e75849-s010.docx]

**Table S1.** Summary of potential harms.

| **Harm Category** | **Primary Risks for Patients** | **Primary Risks for Clinicians** |
| --- | --- | --- |
| **Accuracy** | Misinformation, outdated or hallucinated content leading to poor decisions (e.g., Heart Failure diet error). | Diagnostic errors, inaccurate summaries (e.g., Discharge summary mistake). |
| **Bias** | Biased advice reinforcing stereotypes, worsening inequities. | Biased notes or suggestions, inequitable drafts. |
| **Safety/Care** | Delayed/inappropriate care, false reassurance or alarm, misuse (e.g., replacing professional advice). | Over-reliance, deskilling, missed nuances, workflow issues. |
| **Privacy/Security** | Exposure of sensitive data (e.g., PHI, genetic info), data misuse. | Violations of HIPAA/GDPR, institutional risk, lost trust. |
| **Relationship** | Reduced trust if advice conflicts with providers, misinterpretation. | Depersonalization, communication barriers, trust erosion. |
| **Accountability** | Hard to assess LLM credibility or limitations. | Legal/professional liability unclear for AI-related errors. |

**Table S2.** Examples of harm reduction strategies.

| **User Group** | **Strategy Type** | **Example Implementation (Reflecting Sec 3)** |
| --- | --- | --- |
| **Patients** | Education/Awareness | Clear disclaimers ("Not medical advice"). Guidance on safer prompting. |
|  | Critical Use | Use LLM for background info/questions, verify with provider (e.g., Diabetes questions). |
|  | Verification | Actively verify LLM-suggested resources (e.g., Mental health orgs). |
|  | Technical/UI | Output transparency (confidence scores, source linking – *ideal, often lacking*). |
|  | Policy/Access | Transparency on data privacy; easy opt-out/deletion. |
| **Clinicians** | Education/Training | Training on limitations, bias, safe prompting, critical appraisal. |
|  | Governance/Policy | Institutional guidelines on use (e.g., Drafting vs. Diagnosis). Mandatory human validation. |
|  | Workflow Integration | Review/edit LLM drafts (e.g., Referral letter). Standardized verification protocols. |
|  | Technical/Environment | Use secure, HIPAA/GDPR-compliant enterprise LLMs. Audit trails. |
|  | Specific Use Cases | Use LLMs for low-risk tasks (e.g., Non-clinical comms) or as skeptical aids (e.g., Lit review, Brainstorming). |

**Table S3.** Clinician-patient LLM risk matrix.

| **Risk Category** | **Patients — Typical Impact** | **Clinicians — Typical Impact** | **Overall Residual Risk ᵃ** | **Primary Harm-Reduction Levers** |
| --- | --- | --- | --- | --- |
| **Accuracy / Hallucination** | Unsafe self-management, delayed care (e.g., heart-failure diet error) | Diagnostic anchoring, charting errors | **High** | Mandatory disclaimers; verification prompts for patients; “human-in-the-loop” review & source checking for clinicians |
| **Bias / Inequity** | Reinforced stereotypes → sub-optimal self-care | Biased notes & treatment plans | **High** | Bias-detection tooling; clinician bias-audit checklists; diverse training data; patient literacy resources |
| **Privacy / Security** | PHI leakage, re-identification | Regulatory penalties (HIPAA/GDPR), reputational loss | **High** | End-to-end encryption; enterprise sandbox; explicit data-retention policy; easy user data-deletion |
| **Safety / Care Pathway** | False reassurance or unwarranted alarm | Over-reliance ⇒ deskilling, missed nuances | **Medium–High** | “See-a-professional” nudges; escalation triggers; mandatory clinical validation steps |
| **Relationship / Trust** | Conflicting advice damages trust | Depersonalised communication | **Medium** | Transparent disclosure of LLM use; empathetic framing templates; shared-decision aids |
| **Accountability / Liability** | Confusion over responsibility | Legal uncertainty for adverse outcomes | **Medium** | Clear institutional policy on scope-of-use; audit trails; documented sign-off by clinician |

ᵃ Residual risk after implementing recommended mitigations.

**Table S4.** Governance checklist for safe LLM deployment.

Tick ✓ once each item is in place; review quarterly.

| **Domain** | **Checkpoint** | **✓** |
| --- | --- | --- |
| **Policy & Scope** | • Approved clinical and non-clinical use-cases enumerated • High-risk tasks (diagnosis, prescribing) explicitly prohibited • Disclosure policy to patients defined |  |
| **Technical Controls** | • HIPAA/GDPR-compliant vendor or on-prem model • Role-based access & SSO • PHI redaction or secure context wrapping • Version pinning & update log |  |
| **Training & Competency** | • Mandatory onboarding module (limitations, bias, verification workflow) • Annual competency renewal • Quick-reference prompts library |  |
| **Human-in-the-Loop** | • Named clinician reviewer for every clinical output • Two-step sign-off for discharge summaries/letters • Documented verification of citations/evidence |  |
| **Bias & Fairness** | • Quarterly bias audit of sampled outputs • Diverse stakeholder review board (DEI rep, patient advocate) • Mitigation plan for identified biases |  |
| **Safety & Quality Monitoring** | • Real-time error-report channel • Incident-severity matrix and escalation path • KPIs: error rate, turnaround time, clinician deskilling indicators |  |
| **Privacy & Data Lifecycle** | • Data-retention period defined • User-level “erase my queries” function • Secure logging with anonymisation |  |
| **Regulatory & Legal** | • Liability language in consent forms • Insurance / malpractice carrier notified • Compliance gap analysis updated with new regulations |  |
| **Continuous Improvement** | • Quarterly stakeholder workshop to refine prompts/policies • Feedback loop to vendor or model-ops team • Sunset criteria for under-performing workflows |  |

How to use:
Assign each line-item to a responsible owner.
Track status in the final column (✓/✗/In progress).
Escalate unresolved critical items (>30 days) to the institutional AI governance board.
